# The role of the C8 proton of ATP in the regulation of phosphoryl transfer within kinases and synthetases

**DOI:** 10.1186/1471-2091-12-36

**Published:** 2011-07-13

**Authors:** Colin P Kenyon, Anjo Steyn, Robyn L Roth, Paul A Steenkamp, Thokozani C Nkosi, Lyndon C Oldfield

**Affiliations:** 1 CSIR, Biosciences, Meiring Naude Road, Pretoria, 0001, Gauteng, South Africa

## Abstract

**Background:**

The kinome comprises functionally diverse enzymes, with the current classification indicating very little about the extent of conserved regulatory mechanisms associated with phosphoryl transfer. The apparent *K*_m _of the kinases ranges from less than 0.4 μM to in excess of 1000 μM for ATP. It is not known how this diverse range of enzymes mechanistically achieves the regulation of catalysis via an affinity range for ATP varying by three-orders of magnitude.

**Results:**

We have demonstrated a previously undiscovered mechanism in kinase and synthetase enzymes where the overall rate of reaction is regulated via the C8-H of ATP. Using ATP deuterated at the C8 position (C8D-ATP) as a molecular probe it was shown that the C8-H plays a direct role in the regulation of the overall rate of reaction in a range of kinase and synthetase enzymes. Using comparative studies on the effect of the concentration of ATP and C8D-ATP on the activity of the enzymes we demonstrated that not only did C8D-ATP give a kinetic isotope effect (KIE) but the KIE's obtained are clearly not secondary KIE effects as the magnitude of the KIE in all cases was at least 2 fold and in most cases in excess of 7 fold.

**Conclusions:**

Kinase and synthetase enzymes utilise C8D-ATP in preference to non-deuterated ATP. The KIE obtained at low ATP concentrations is clearly a primary KIE demonstrating strong evidence that the bond to the isotopically substituted hydrogen is being broken. The effect of the ATP concentration profile on the KIE was used to develop a model whereby the C8H of ATP plays a role in the overall regulation of phosphoryl transfer. This role of the C8H of ATP in the regulation of substrate binding appears to have been conserved in all kinase and synthetase enzymes as one of the mechanisms associated with binding of ATP. The induction of the C8H to be labile by active site residues coordinated to the ATP purine ring may play a significant role in explaining the broad range of *K*_m _associated with kinase enzymes.

## Background

The International Union of Pure and Applied Chemistry and the International Union of Biochemistry (IUPAC/IUB) commission on the classification and nomenclature of enzymes placed the enzymes that transfer high energy phosphate bonds from nucleotides into two divisions: the transferases (kinases) and ligases (synthetases) [1].

The transferases have been placed in Division 2 and the ligases into Division 6 of the Enzyme Commission (EC) classification. The ligases are enzymes catalysing the joining of two molecules with the concomitant hydrolysis of the pyrophosphate bond of ATP, while a kinase is defined as an enzyme which catalyses the transfer of the phosphate group from ATP (or GTP) to a substrate containing an alcohol, amino, carboxyl, or phosphate group as the phosphoryl acceptor [2,3]. The kinases are a large number of structurally diverse enzymes that play a critical role in numerous metabolic and signalling pathways and whose substrates may be a small molecule, lipid, or protein. They have been classified into 25 families of homologous proteins, with the families assembled into 12 fold-groups based on the similarity of their structural folds [2]. However, this classification relays little, if any, information on the catalytic and regulatory mechanisms employed in nucleotide binding and phosphoryl transfer. Within a single group, both prokaryotic and eukaryotic organisms are represented with kinase isoenzymes that appear to be kinetically and functionally distinct based on the rate of phosphoryl transfer and the regulation thereof.

This investigation was undertaken to ascertain the extent to which the adenyl group within ATP plays a direct role in the regulation of ATP binding and/or phosphoryl transfer within a range of kinase and synthetase enzymes. To this end the role of the C8-H of ATP on the binding and/or phosphoryl transfer on the enzyme activity of a number of kinase and synthetase enzymes was elucidated in comparative enzyme activity essays using ATP and ATP deuterated at the C8 position. Kinetic isotope effects (KIE) are broadly classified into primary, secondary and stearic effects. The extent of proton/deuterium KIE's is estimated from the rate constants (KIE = *v*_H_/*v*_D_) and a KIE of the order of 2 or more is strong evidence that the bond to the isotopically substituted hydrogen atom is being broken in the rate determining step of the reaction for the primary KIE and are as a result of bond breaking [4,5]. The calculated maximum for the KIE involving C-H bonds is approximately 7 at room temperature as determined by the difference in the zero-point energy-difference between the bond to the deuterium and the bond to the hydrogen . The secondary deuterium KIE's are defined as the isotope effect when the bond to the isotopically substituted atom is not cleaved but occur as a result of a hybridization change. Secondary KIE's are classified as α or β, depending on whether the isotopic substitution is made on the α or β centre relative to the atom undergoing the chemical change or further away within the molecule. If the hybridization change is from sp^3 ^to sp^2^, the KIE is normal however if the hybridization change is sp^2 ^to sp^3 ^the KIE is inverse, with the range being 1.4 to 0.7, respectively. Stearic effects also affect the KIE to the same extent as the secondary KIE's.

In oligomeric enzymes it is proposed that the deuteration of ATP not only affects the binding of ATP to the site where catalysis is occurring but the deuteration also affects the interaction between sites. In oligomeric kinases it is proposed that mechanistically two modes of regulation occur, one which is dependent on the release of ADP from the first active site before ATP binds to the second active site and the second mode of regulation depends on the conversion of ATP to ADP prior to the binding of the ATP to the second active site. To this end a range of kinase and synthetase enzymes were tested to assess the role of the C8-H of ATP in the binding and steady state enzyme activity of these enzymes.

Acetate kinase (EC 2.7.2.1) is a homodimer which catalyses the Mg^2+^-dependent, reversible transfer of phosphate from ATP to acetate in the following reaction:

Acetate kinase forms part of the acetate and sugar kinase/Hsc70/actin (ASKHA) structural superfamily (PFam Clan: *Actin_ATPase*:CL0108) [6]. The enzyme is a homodimer and monomer interaction plays a role in the regulation of the enzyme activity and ligand binding with the enzyme active sites functioning in a coordinated half-the-sites manner [7-9]. The actin ATPase clan contains both the acetate kinases and sugar kinases and are all known to undergo a catalytically essential domain closure upon ligand binding.

Hexokinase (ATP: D-hexose 6-phosphotransferase, EC 2.7.1.1) catalyses the Mg^2+^-dependent phosphorylation of glucose, from ATP:

The two isoenzymes of yeast hexokinase, designated P-I and P-II, are dimers of subunit molecular mass 52 kDa [10-15]. Hexokinase also forms part of the acetate and sugar structural superfamily (PFam Clan: *Actin_ATPase*:CL0108). Yeast hexokinase enzymes are structurally well characterised with each subunit of the homodimer comprising two domains and in the open conformation these domains are separated by a cleft containing the sugar binding site [16-22]. Binding of glucose induces a large conformational change in which the two lobes of the subunit rotate relative to each other. The enzymes also exist in a monomer-dimer association-dissociation equilibrium that is influenced by pH, ionic strength and substrates. There are major differences in the glucose binding behaviour of both forms where binding to dimeric P-I shows strong positive cooperativity, whereas in P-II the two sites are equivalent and binding is non-cooperative [23-26].

The shikimate pathway is a seven-step biosynthetic route that links the metabolism of carbohydrates to the synthesis of aromatic amino acids via the conversion of erythrose-4-phosphate to chorismic acid [27]. Shikimate kinase (SK, EC 2.7.1.71), the fifth enzyme in the shikimate biosynthetic catalyzes phosphate transfer from ATP to the carbon-3-hydroxyl group of shikimate, forming shikimate 3-phosphate. SK belongs to the nucleoside monophosphate (NMP) kinase structural family where the characteristic feature of the NMP kinases is that they undergo large conformational changes during catalysis and belongs to the P-loop containing nucleoside tri-phosphate hydrolase superfamily (Pfam Clan: *AAA*:CL0023) [28]. The NMP kinases are composed of three domains. The CORE contains a highly conserved phosphate-binding loop (P-loop), the LID domain, which undergoes substantial conformational changes upon substrate binding, and the NMP-binding domain, which is responsible for the recognition and binding of a specific substrate [29]. The SK crystal structures show that SK exists as a monomer with a single ATP binding site and MgADP induces concerted hinged movements of the shikimate binding and LID domains causing the two domains to move towards each other in the presence of this ligand [29].

Phosphofructokinase (PFK, fructose-6-phosphate 1-kinase, EC 2.7.1.11) is a classical allosteric enzyme that catalyzes the phosphorylation of D-fructose 6-phosphate (Fru-6-P) by Mg-ATP to form D-fructose 1,6-bisphosphate and MgADP. PFK from *B. stearothermophilus *is a homo-tetramer with each subunit having a molecular weight of 34 kDa, which undergoes a concerted two-state allosteric transition [30]. PFK belongs to the PFK-like superfamily (Pfam Clan: *PFK*:CL0240) The enzyme from *B. stearothermophilus *(Bs-PFK) shows hyperbolic Michaelis-Menten kinetics with respect to both Fru-6-P and Mg-ATP, but cooperative kinetics in the presence of allosteric inhibitor phosphoenolpyruvate(PEP) [31]. Unliganded Bs-PFK is in the active R state, which has high affinity for substrate, switching to the inactive T state with low affinity for substrate only in the presence of PEP.

Glutamine synthetase (GS) (EC 6.3.1.2) catalyzes the reversible conversion of L-glutamic acid, ATP and ammonia to L-glutamine, ADP and inorganic phosphate via a γ-glutamyl phosphate intermediate [32]. As GS is a central enzyme in nitrogen metabolism the enzyme is regulated by at least four different mechanisms: (a) adenylylation and deadenylylation of the tyrosine 397 residue, (b) conversion between a relaxed (inactive) and taut (active) state depending on the divalent metal cation present, (c) cumulative feedback inhibition by multiple end products of glutamine metabolism, and (d) repression and derepression of GS biosynthesis in response to nitrogen availability [32]. *Escherichia coli *GS is a large, metalloenzyme (~624 kDa) comprising 12 identical subunits arranged in two face-to-face hexagonal rings [33].

*E. coli *GS belongs to the glutamine synthetase 1-β group of enzymes that are regulated via adenylylation of a single tyrosine residue [34], with each subunit requiring two structurally implicated divalent cations (either Mg^2+ ^or Mn^2+^) for its catalytic activity.

The extent of adenylylation of the *E. coli *GS in response to an excess or deficiency of nitrogen in the growth environment is regulated in response to the intracellular concentrations of 2-ketoglutarate and glutamine, via the reversible adenylylation of a tyrosine residue (Tyr397) in each subunit of GS [32,35-39]. The presence of adenylylated GS (GS_12_) predominates in a nitrogen-rich, carbon-limited media, while the deadenylylated form (GS_0_) tends to predominate under conditions of nitrogen limitation [32,35-46].

**Figure 1:**
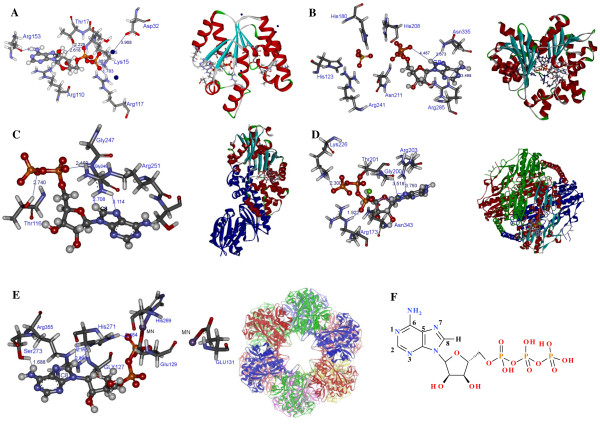
**Coordination of ADP and ATP**. The coordination of either ADP or ATP in the active sites of (A) shikimate kinase (Accession number, 1L4U), (B) acetate kinase (Accession number, 1TUY), (C) hexokinase (Accession number, 2E2P), (D) phosphofructokinase (Accession number, 3F5M), (E) glutamine synthetase (Accession number, 1F52), and (F) ATP atom numbering. The C8H of the ADP in the active site of shikimate kinase (A) is coordinated to the oxygen of the α-phosphate via Thr17 via a hydrogen bonding network. The C8H of the ADP in the active site of acetate kinase (B) is coordinated to the oxygen of the β-phosphate however, the di-phosphate of the ADP is orientated away from the SO_4_^2- ^which occupies the acetate binding site indicating a distortion in the conformation of the ADP within the active site. The C8H of the ADP is within hydrogen bonding distance of Arg 285 and Asn 335. The C8H of the ADP in the active site of hexokinase kinase (C) is coordinated to the oxygen of the α-phosphate via the guanidium moiety of Arg25. The C8H of the ADP in the active site of phosphofructokinase (D) is directly coordinated to the oxygen of the α-phosphate. The C8H of the ATP in the active site of glutamine synthetase (E) impact on the coordination of the β-phosphate via the coordination to the backbone carbonyl of His271. All inter-atomic distances are as indicated.

Comparative steady-state enzyme activity assays were run to determine the effect of ATP and C8D-ATP on the specific activity of a range of kinase and synthetase enzymes. The reason for the investigation was to establish whether the C8H of ATP plays a primary role in the binding of ATP. The enzymes investigated were hexokinase, acetate kinase, shikimate kinase, phosphofructokinase, adenylylated (GS_12_) and deadenylylated GS (GS_0_). The coordination of ADP/ATP in the active sites of the enzymes under investigation, showing the interactions of the C8H with the active site, is outlined in Figure 1. It was demonstrated that the enzymes selectively bind C8D-ATP at low concentrations giving a KIE in excess of 2. It was also demonstrated that if given a combination of ATP and C8D-ATP the enzymes preferentially utilised the C8D-ATP.

## Results

### Effect of C8D-ATP on the specific activity

**Figure 2:**
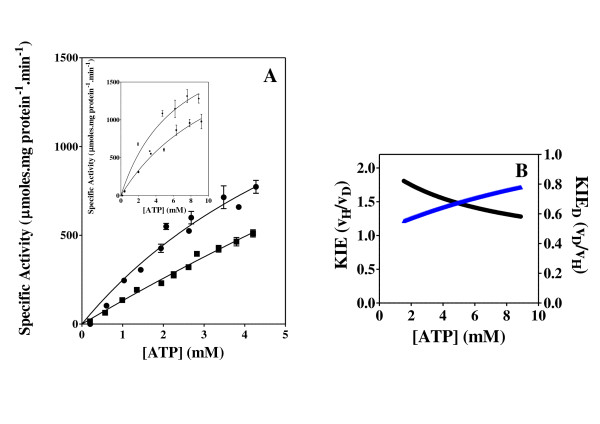
**Shikimate kinase activity and KIE**. Effect of the concentration of ATP and C8D-ATP on the specific activity (A) and KIE (B) of *Mycobacteria tuberculosis *shikimate kinase. (A) "black circle " = ATP, black square" = C8D-ATP, (B) Black = KIE, Blue = KIE_D_. The specific enzyme activity is expressed as moles ADP formed per minute per milligram protein.

**Figure 3:**
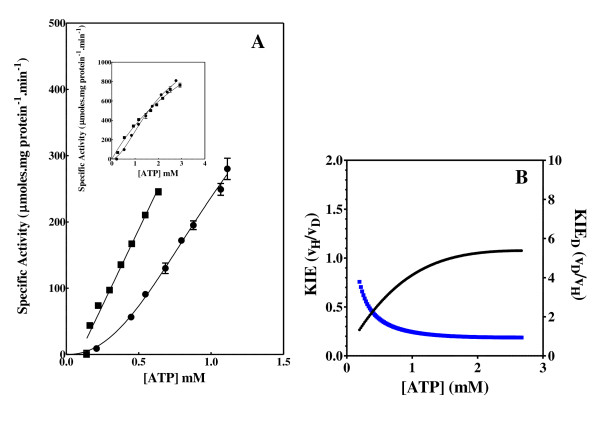
**Hexokinase activity and KIE**. Effect of the concentration of ATP and C8D-ATP on the specific activity (A) and KIE (B) of *Saccharomyces cerevisiae *hexokinase. (A) "black circle " = ATP, black square" = C8D-ATP, (B) Black = KIE, Blue = KIE_D_. The specific enzyme activity is expressed as moles ADP formed per minute per milligram protein.

**Figure 4:**
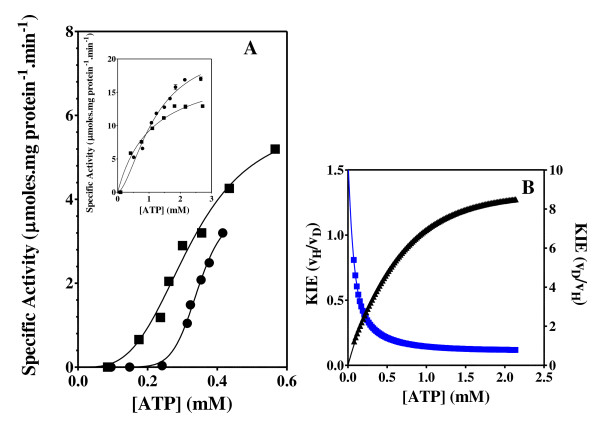
**Acetate kinase activity and KIE**. Effect of the concentration of ATP and C8D-ATP on the specific activity (A) and KIE (B) of *E. coli *acetate kinase. (A) "black circle " = ATP, black square" = C8D-ATP, (B) Black = KIE, Blue = KIE_D_. The specific enzyme activity is expressed as μmoles ADP formed per minute per milligram protein.

**Figure 5:**
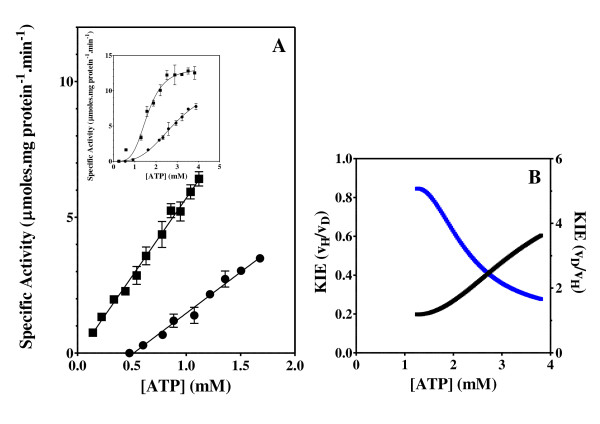
**Deadenylylated glutamine synthetase activity and KIE**. Effect of the concentration of ATP and C8D-ATP on the specific activity (A) and KIE (B) of *Escherichia coli *GS_0_. (A) "black circle " = ATP, black square" = C8D-ATP, (B) Black = KIE, Blue = KIE_D_. The specific enzyme activity is expressed as moles ADP formed per minute per milligram protein.

**Figure 6:**
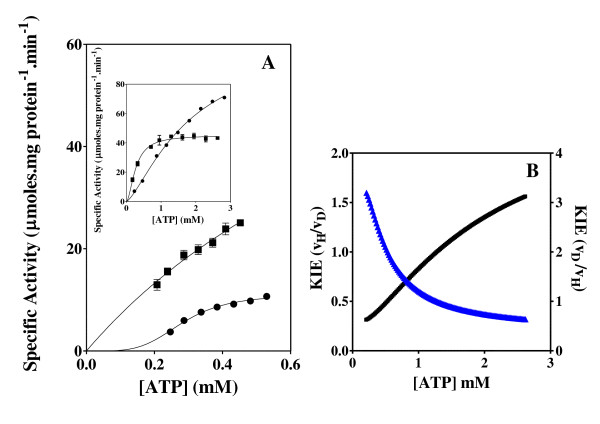
**Phosphofructokinase activity and KIE**. Effect of the concentration of ATP and C8D-ATP on the specific activity (A) and KIE (B) of *B. stearothermophilus *phosphofructokinase. (A) "black circle " = ATP, black square" = C8D-ATP, (B) Black = KIE, Blue = KIE_D_. The specific enzyme activity is expressed as moles ADP formed per minute per milligram protein.

**Figure 7:**
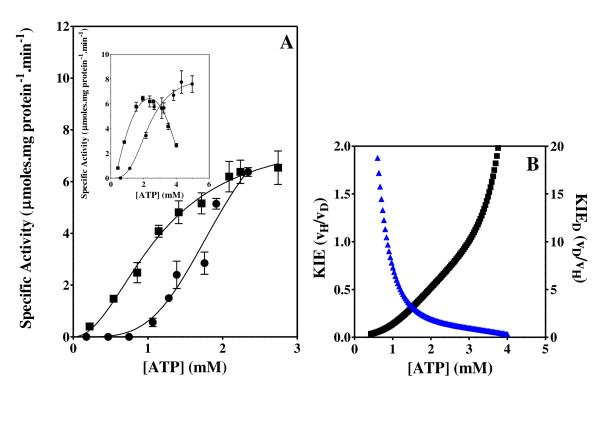
**Adenylylated glutamine activity and KIE**. Effect of the concentration of ATP and C8D-ATP on the specific activity (A) and KIE (B) of *Escherichia coli *adenylylated glutamine synthetase. (A) "black circle " = ATP, black square" = C8D-ATP, (B) Black = KIE, Blue = KIE_D_. The specific enzyme activity is expressed as moles ADP formed per minute per milligram protein.

**Figure 8:**
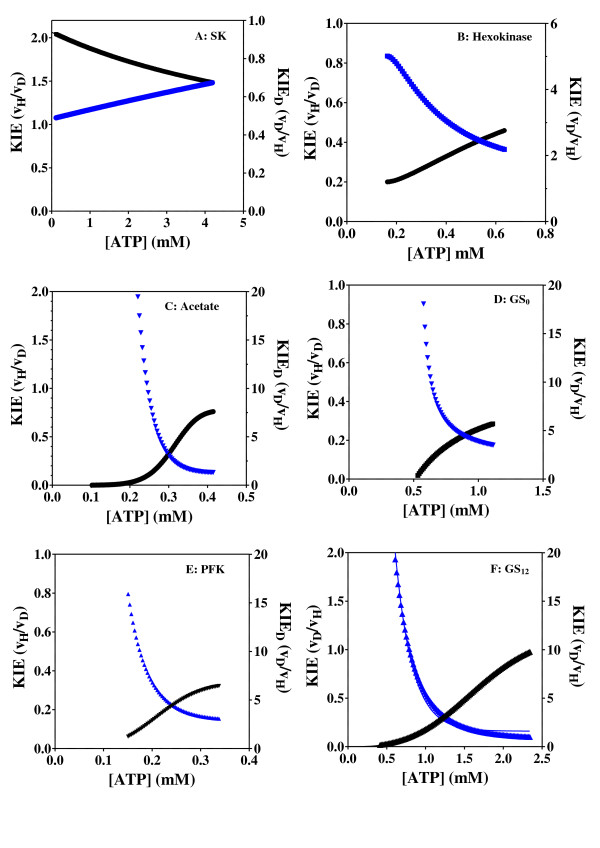
**Effect of low ATP concentrations on the KIE**. Effect of low concentrations of ATP and C8D-ATP on the KIE and KIE_D _obtained for A: shikimate kinase, B: hexokinase, C: acetate kinase, D: GS_0_, E: PFK and F: GS_12_. Black = KIE, Blue = KIE_D_. The specific enzyme activity is expressed as moles ADP formed per minute per milligram protein.

The effect of the ATP and C8D-ATP concentration on the steady state specific activity of *Saccharomyces cerevisiae *hexokinase, *Escherichia coli *acetate kinase, *Escherichia coli *phosphofructokinase, *Escherichia coli *deadenylylated glutamine synthetase (GS_0_), *Escherichia coli *adenylylated glutamine synthetase (GS_12_) and *Mycobacterium tuberculosis *shikimate kinase was determined (Figures 2, 3, 4, 5, 6, 7, 8). Where possible the effect of the ATP and C8D-ATP on the specific activity of the enzyme was expressed over a concentration profile that included the ATP or C8D-ATP concentrations that tended towards *v*_max _as well as an ATP or C8D-ATP concentration profile at low concentrations that would allow for the accurate determination of the KIE. The best-fit to the data was obtained for the specified kinetic model using the non-linear regression algorithms as outlined using the GraphPad Prism^® ^5 software (Table 1). The basis for the selection of the specified models is outlined in the Additional Files 1 (Table S1A & S1B). As part of the software output a data-table was created containing 150 data-points defining the best kinetic fit for each enzymes response to the presence of either ATP or C8D-ATP. These response curves were then used to define the KIE by the conventional estimation of KIE from KIE = *v*_H_/*v*_D_. The inverse KIE (KIE_D_) was also determined using the following function:

**Table 1:** Effect of the concentration of ATP and C8D-ATP on the fit of the enzyme kinetic model of hexokinase, acetate kinase, adenylylated GS, deadenylylated GS, phosphofructokinase and shikimate kinase.

Enzyme	ATP : Kinetic model	C8D-ATP: Kinetic model	KIE_*v*max_
**Shikimate kinase**	Michaelis-Menton	Michaelis-Menton	1

***v*_max_**	2339 ± 600	2433 ± 600	
***K*_m _or *K*'**	0.0065 ± 0.003	0.0128 ± 0.007	
**RMSD^a^**	0.9543	0.9866	
**Hexokinase**	Allosteric sigmoidal	Allosteric sigmoidal	1

***v*_max_**	1226 ± 189	1893 ± 940.7	
***K*_m _or *K*'**	3.263 ± 0.662	4.419 ± 2.749	
**h**	1.759 ± 0.207	1.020	
**RMSD^a^**	0.9959	0.9919	
**Acetate kinase**	Allosteric sigmoidal	Michaelis-Menton	1

***v*_max_**	22.29 ± 3.046	19.13 ± 2.103	
***K*_m _or *K*'**	1.323 ± 0.353	1.081 ± 0.289	
**h**	1.658 ± 0.302		
**RMSD^a^**	0.9880	0.9704	
**Deadenylylated GS**	Allosteric sigmoidal	Allosteric sigmoidal	1

***v*_max_**	11.49 ± 1.31	13.20 ± 0.651	
***K*_m _or *K*'**	30.88 ± 4.44	6.774 ± 2.331	
**h**	3.108 ± 0.316	4.077 ± 0.787	
**RMSD^a^**	0.9972	0.9827	
**PFK**^b^	Allosteric sigmoidal	Allosteric sigmoidal	2

***v*_max_**	111.3 ± 10.16	45.18 ± 1.012	
***K*_m _or *K*'**	2.265 ± 0.320	0.097 ± 0.031	
**h**	1.371 ± 0.098	1.785 ± 0.208	
**RMSD^a^**	0.9982	0.9879	
**Adenylylated GS**	Allosteric sigmoidal	Allosteric sigmoidal	>2

***v*_max_**	8.241 ± 0.787	6.540 ± 0.271	
***K*_m _or *K*'**	15.22 ± 8.776	0.603 ± 0.151	
**h**	3.288 ±	3.258 ± 0.589	
**RMSD^a^**	0.9822	0.9924	

The response of each enzyme to change in the ATP and C8D-ATP concentration was tested for the fit to either an allosteric sigmoidal model or to the Michaelis-Menton model of enzyme kinetics by non-linear regression using the GraphPrism 5 software. The *K*_m _or *K*' were estimated depending on the model. The root mean square deviation of the data from the model is as outlined. The Hill factor for the allosteric sigmoidal model is as indicated. KIE_*v*max _is equal to the KIE attained at ATP and C8D-ATP concentrations at maximum enzyme activities.

^a ^Root mean square deviation of the data defining the kinetic model

^b ^Phosphofructokinase

Where *v*_D _= specific activity in the presence of C8D-ATP

*v*_H _= specific activity in the presence of ATP.

The calculation of KIE_D _was used as the extent of the KIE_D _is instructive in the putative role that the C8H of ATP plays in the regulation of phosphoryl transfer as in the case of the oligomeric enzymes the activity in the presence of C8D-ATP was higher at low concentrations then that obtained using ATP. The calculation of KIE_D _therefore gave integer values in excess of 1 which may be indicative of the mode of allosteric regulation found in oligomeric enzymes.

In all 6 cases defined a KIE was obtained in response to presence of C8D-ATP and in all cases other than shikimate kinase the KIE_D _at low ATP concentrations is in excess of 5 (Figures 2, 3, 4, 5, 6, 7, 8). In monomeric enzymes, such as shikimate kinase, as the concentration of ATP and C8D-ATP was increased there appears to be concomitant increase in the KIE_D _while in oligomeric enzymes there is a decrease in the KIE_D _with increasing ATP concentrations (Figure 2, 3, 4, 5, 6, 7, 8). The KIE obtained was a primary KIE as the extent of the KIE was two-fold or significantly in excess of two-fold at low concentrations. The KIE_D _over the full ATP/C8D-ATP concentration range appeared to be indicative of the mode of regulation of the enzyme as in all cases the KIE either positively or negatively asymptotes to a specific constant integer value. The KIE of shikimate kinase asymptotes negetively to a KIE of 1.0 as the specific activity tends towards *v*_max_. This is a classical KIE effect with the KIE being 2 at low ATP concentrations, asymptoting to a level of 1 (Figure 2, Table 1). Shikimate kinase exists as a monomer and therefore no regulation occurs via the interaction of the subunits that may affect the overall KIE. Hexokinase, acetate kinase and GS_0 _appear to use the same mechanism for regulation with the KIE_D _of these enzymes negatively asymptoting to 1 at *v*_max _(Figures 3, 4, 5, Table 1). All three of these enzymes are multi-meric and allosteric regulation may occur via the interaction of subunits. The hexokinase and acetate kinase are both homodimers and monomer interaction plays a role in the regulation of the enzyme activity and ligand binding with the enzyme active sites functioning in a coordinated half-the-sites manner [7-9]. Phospho-fructokinase and GS_12 _use a similar mechanism with the KIE_D _asymptoting to a level of 0.5 at *v*_max _(KIE = 2) (Figures 6, 7; Table 1). *E. coli *GS_12 _is a dodecamer consisting of two stacked hexameric structures consisting of 12 identical subunits. The subunits probably interact allosterically on the binding of ATP as occurs in phosphofructokinase. The slow rate of release of C8D-ADP from the interacting active site of GS_12 _probably impacts on the binding of ATP in the adjacent site.

### Selectivity for C8D-ATP

**Figure 9:**
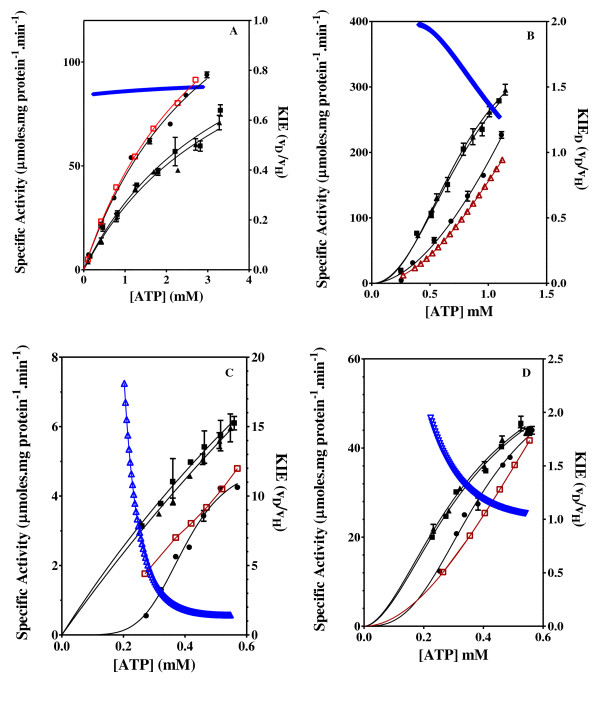
**Effect of ATP, C8D-ATP and ATP/C8D-ATP (1:1 ratio) concentrations**. Effect of ATP, C8D-ATP and ATP/C8D-ATP (1:1 ratio) concentrations on the specific activity and KIE of shikimate kinase (A), hexokinase (B), acetate kinase (C) and PFK (D). The theoretical activity was obtained using the constants and the best model defining each enzyme activity profile for ATP and C8D-ATP to estimate the activity at each concentration assuming mix of ATP/C8D-ATP. The activity estimated for ATP and C8D-ATP was summed to determine the activity for ATP/C8D-ATP. "black circle " = ATP, black square" = C8D-ATP, "black triangle" = ATP/C8D-ATP, Red = Theoretical activity, Blue = KIE_D_. The specific enzyme activity is expressed as moles ADP formed per minute per milligram protein.

The selectivity of a number of kinases for C8D-ATP was determined using the steady state enzyme activity in the presence ATP, C8D-ATP and assays containing ATP and C8D-ATP in a 1:1 ratio equivalent to the total concentration used in the ATP and C8D-ATP assays (Figure 9). In all cases the enzymes appear to have a preference for C8D-ATP. In the case of the oligomeric kinases, namely acetate kinase, hexokinase and phosphofructokinase the enzymes have a greater affinity for C8D-ATP than ATP as the activity obtained in the presence of the combination of ATP and C8D-ATP at a 1:1 ratio was significantly higher than in the case of the ATP and the enzyme activity profile of the assay containing the combination followed that of the C8D-ATP. In the case of the shikimate kinase the activity obtained in the presence of the combination of ATP and C8D-ATP was similar to the steady state enzyme activity obtained in the presence of deuterated ATP on its own. The shikimate kinase, which has a classical kinetic isotope effect, appears to selectively utilize the C8D-ATP when assayed in the presence of both ATP and C8D-ATP as the enzyme activity profile follows that of the C8D-ATP. However, this may be as a result of the difference in the binding equilibria for ATP and C8D-ATP (see discussion). High resolution mass spectroscopy (MS) of assay solutions demonstrated there is in fact a preference for ATP by shikimate kinase and C8D-ATP by the oligomeric enzymes. At low ATP concentrations the relative intensities of the C8D-ADP to ADP peaks were higher in the case of the oligomeric enzymes indicating a preference for C8D-ATP in assays run at low a ATP concentration, while the reverse was true for shikimate kinase (Additional files 1, Figure S2B, S3B, S4B). The relative intensities obtained from assays run at the high concentrations were equivalent also indicating a KIE of 1 (Additional files 1, Figure S2B, S3B, S4B). In other words the mass spectroscopy data gave similar trends as was obtained for the enzyme activity data. If the assays are run to achieve too great an ATP/C8D-ATP conversion this effect would be negated in part as a result of the C8D-ATP being selectively utilized and the relative concentration of ATP to C8D-ATP in solution would increase thereby compensating for the deuteration effect by the concentration effect.

## Discussion

**Figure 10:**
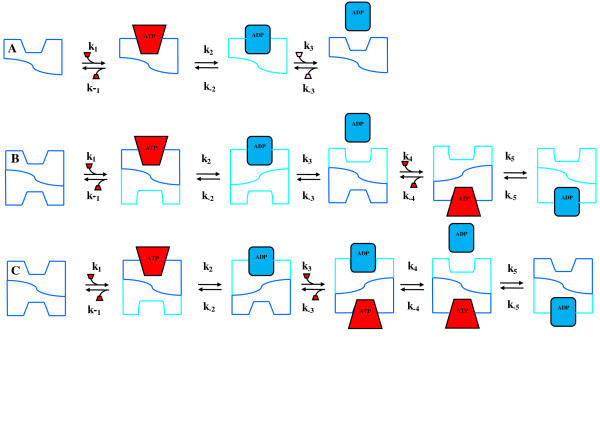
**Models for the binding of nucleotides to kinases and synthetase enzymes**. Models for the binding of nucleotides to kinases and synthetase enzymes. A. Model for the binding of ATP and release of ADP from monomeric kinase. The current model for oligomeric kinases is based on nonequivalent ligand binding where binding to one monomer affects binding to a second monomer, or coordinated active sites. B. Model based on the rate reaction in the conversion of ATP to ADP with the concomitant conversion of the second binding site to the ATP-binding-form as a result of the release of the ADP. Once the ADP has been released from the first site this changes the affinity of the second site from an ADP binding structure to an ATP binding structure. C. Model based on the rate reaction in the conversion of ATP to ADP with the concomitant conversion of the second binding site to the ATP-binding-form as a result of the conversion of ATP to ADP in the first binding site. Once the ATP is converted ADP this changes the affinity of the second site from an ADP-binding structure to an ATP-binding-form.

The role of the KIE in the kinetics of the enzymes investigated has led to models for the regulation of the binding of ATP to be proposed (Figure 10). In classical steady-state kinetics as represented by the Briggs-Haldane modification of the Michaelis-Menton formulation (Equation 1),(1)

and *k*_on _= *k*_1_, *k*_off _= *k*_-1 _and *k*_2 _>>*k*_-1_, and the Michaelis constant, *K*_M _is obtained from(2)

Where E, S and P are the enzyme, substrate (ATP) and product (ADP) concentrations respectively. In monomeric enzymes such a shikimate kinase the effect of the concentration of ATP and C8D-ATP on the relative enzyme activities results in the KIE asymptoting from 2 to 1. If the C8H of ATP is not directly implicated in phosphoryl transfer, the deuteration of ATP may affect the equilibrium of the reaction either by affecting the binding of ATP, *k*_1_, or by the release of ADP, *k*_3_. In monomeric enzymes which follow Michaelis-Menton kinetics, *K*_M _is dependent on *k*_1_, *k*_2 _and *k*_3_. If the C8D impacts directly on the overall reaction mechanism then the KIE is as a result of breaking the bond to the isotopically labelled atom. The regulation may however, be dependent on the equilibrium of binding of ATP, *k*_1_, or the equilibrium of release of ADP, *k*_3_, also being implicated in the breaking and re-making to the C8H bond. The regulation of the reaction rate by both C8D-ATP *per se *and the concentration of C8D-ATP or ATP probably arise as a result of the same equilibrium constant, either *k*_1 _or *k*_3_. At low ATP concentrations the deuteration of C8 may result in the retardation of the release of ADP from the active site and at high ATP concentrations the concentration effect of ATP results in a reduction in the rate of release of ADP from the active site. Due to the magnitude of the KIE it would appear that bond-breaking of C8H(D) is implicated in the binding of ATP and release of ADP. The impact on *k*_3 _does not appear to be the cause of the high KIE at low concentrations where the KIE is the largest. The effect of the increase in the ATP/C8D-ATP concentration on the KIE therefore only manifests as the classical effect with the KIE being of the order of 2.0 as determined by *v*_H_/*v*_D_, at low concentrations, asymptoting to 1 at high ATP concentrations where both concentration and the role of C8D impact on the KIE. At low concentrations of ATP the enzyme activity is dominated by the impact of the C8H/C8D on the equilibrium of binding. At low concentrations the C8H plays the predominant role in the equilibrium of binding as in the case of shikimate kinase if the enzyme is exposed to a mixture of ATP and C8D-ATP, where the activity follows that of the C8D-ATP (Figure 9). The *K*_M _for ATP is however lower than for C8D-ATP (Table 1). If *k*_1 _for ATP is greater than *k*_1 _for C8D-ATP the response seen in the mixture would manifest similar to a reduction in the concentration of ATP thereby reducing the overall rate of reaction. At low ATP concentrations the mass spectroscopy data in fact indicates that the ATP is preferentially utilised as seen by the relative concentrations of ADP in the assay solutions whereas at high concentrations the relative concentrations of ATP and C8D-ATP are equivalent (Additional files 1, Figure S1B). At low ATP concentrations *k*_1 _(*k*_on_) predominates and as the concentration of ATP increases the concentration effect plays an increasing role in the KIE thereby negating the impact of the C8H on the KIE. The reduction in the overall enzyme activity by C8D-ATP may be as a result of the breaking of the C8D bond having a direct impact on *k*_2_, the phosphoryl transfer reaction. As the classical H/D KIE is of the order of 2, as the concentration of ATP tends towards the concentration at the maximum specific activity, *v*_max_, where the concentration effect is at its maximum the effect of the C8H/C8D on the KIE is at a minimum and the KIE tends towards 1.

In oligomeric enzymes it is proposed that the deuteration of ATP not only affects the binding of ATP to the site where catalysis is occurring but the deuteration also affects the interaction between sites. In oligomeric kinases it is proposed that mechanistically two modes of regulation occur, one which is dependent on the release of ADP from the first active site before ATP binds to the second active site (Figure 10B) and the second mode of regulation depends on the conversion of ATP to ADP prior to the binding of the ATP to the second active site (Figure 10C). In the mechanism outlined in Figure 10C binding to the second site can occur prior to the release of ATP from the first site provided the reaction from ATP to ADP has occurred.

It is proposed in enzymes such as acetate kinase, hexokinase and GS_0_, which utilise the coordinated half-sites mechanism of regulation, the enzyme kinetics follows classical allosteric kinetics where an equilibrium is set up between the enzyme concentration [E] and the substrate concentration [S] and binding of the second ATP is dependent on the conversion of the second active site into an ATP binding form by the release of ATP from the first active site. In enzymes using this mechanism of regulation, *K*_M _is dependent primarily on *k*_-1_, *k*_2 _and *k*_3_. The KIE obtained in these enzymes asymptotes to a value of 1 from low levels (KIE_D _values asymptote to levels significantly above 2). At low ATP concentrations the effect of the deuteration of C8 is to allow binding to occur for long enough to allow the reaction to occur and negate the effect of *k*_-1_, thereby shifting the equilibrium to *k*_2_. At low ATP concentrations therefore the impact of the deuteration on the binding is to retard the release of the ATP. At high ATP concentrations the impact of the ATP concentration relative to the impact of ATP binding on the rate of reaction is significantly higher and as a result there is a concomitant increase in the KIE. The impact of binding and the reaction rate however equilibrate to a KIE of 1. The maximum rate of binding can only ever be equivalent to the maximum rate at which the second ATP binding site is converted to the ATP binding form by the release of ADP from the first site (Figure 10B). The inverse KIE, KIE_D_, at low ATP concentrations is as a result of an increase in the probability of the reaction occurring resulting from the deuteration and not as a result of activation of binding *per se*, as in the half-sites-activity mechanism there is no activation of the activity of the second site as a result of the interaction with the first site. The classical impact of deuteration on the KIE when the KIE is a primary effect, as determined by *v*_H_/*v*_D_, should yield a KIE of 2 or more [4,5]. As the regulation of the enzyme activity and ligand binding in these enzymes function in a coordinated half-the-sites manner binding in the second site only occurs on release of the ADP from the first site, it is therefore proposed that deuteration of the ATP improves the binding characteristics. As the equilibrium shifts towards the impact of increasing ATP concentration on the enzyme activity the deuterated ATP binds effectively twice as efficiently as the non-deuterated ATP thereby negating the impact of the deuteration on the apparent enzyme activity at high ATP concentrations, yielding a KIE of 1.

In enzymes where the second active site is made amenable to ATP binding by the conversion of ATP to ADP, in other words binding may occur to the second site prior to the release of the ATP from the first site, the *K*_M _is dependent on *k*_1 _and *k*_2_. This occurs in the case of phosphofructokinase and GS_12 _where the KIE becomes 2 at *v*_max _(Figure 6 &7). The impact of this binding is that at any point in time up to two or more reactions might be occurring simultaneously in two active sites. Unlike in the half-sites mechanism in these enzymes activation of the activity in the second site (or more) might occur from the first site. The binding *per se *therefore might have an effect on the KIE_D_. In multimeric enzymes this effect might be greater. At high concentrations the deuterated ATP binds twice as efficiently as the non-deuterated ATP this allows the KIE to asymptote to 2 or more. At low concentrations the deuteration has the same effect as occurs in the previous model, whereby binding occurs for a long enough period to negate the effect of *k*-_1_. At high concentrations the effect of deuteration is superseded by the concentration effect and as two or more active sites are able to function simultaneously, this allows the KIE to asymptote to 2 or more. It is proposed that a result of the adenylylation of GS it allows for the regulation of the enzyme by a similar mechanism as occurs in phosphofructokinase. Bacterial PFK is a homoteramer, with the four subunits assembled as a dimer of dimers [47-49]. It is conceivable that on adenylylation of GS the interaction between two-subunits effectively creates a dimer of dimer interaction.

## Conclusions

The data outlined clearly demonstrates the role of C8H of ATP in the kinetics and regulation of a number of kinase and synthetase enzymes. The KIE is clearly a primary KIE. However, the extremely high values of the KIE_D _obtained at low at concentrations in the case of the oligomeric enzymes does not appear to be as a result of the impact of the deuterium on the rate the phosphoryl transfer mechanism *per se*, but rather as a result of the role that the C8H plays in the equilibrium of binding of the ATP to the active site. Clearly the regulation of enzyme activity in kinases and synthetases is complex, which manifests in the apparent *K*_M _of the kinases ranging from less than 0.4 μM to in excess of 1000 μM for ATP (Carna Biosciences, Inc., Kinase Profiling Book:http://www.carnabio.com). The findings of this investigation have demonstrated that the C8H of ATP plays a direct role in binding of ATP to the active site of enzymes. The labile nature of the C8H of ATP is well documented [50-52]. It is therefore conceivable that the role of the C8H of ATP in the kinetics and regulation of enzyme activity has been conserved in all kinase and synthetase enzymes as one of the regulatory mechanisms associated with binding of ATP to the active site of this diverse range of enzymes. The induction of the C8H to be labile by active site residues coordinated to the ATP purine ring may play a significant role in explaining the broad range of *K*_m _associated with kinase enzymes.

The exact role of the C8H in the stabilization of the ATP-substrate transition state is unclear. All kinase and synthetase enzymes have an absolute requirement for the presence of a divalent metal ion, either Mg^2+ ^Mn^2+^, for enzyme activity. The principal effect of the metal ion is to facilitate the nucleophilic attack by charge neutralization. It is conceivable that the labile proton also plays a role in the transient redistribution of charge during nucleophilic attack. It has been demonstrated that ATP analogues substituted at the C8 position significantly decrease the affinity of the analogues for cAMP-dependent protein kinase. As large molecular volume substitution at the C8 position leads to compounds existing primarily in the *syn*-conformation the data lead to the conclusion that ATP preferentially binds in the *anti*-conformation [53]. Competitive binding and inhibition of Na, K-ATPase was also obtained using ATP analogues substituted at the C8 position [54]. Na, K-ATPase was found to be able to hydrolyze a number of the C8-substituted analogues having unaltered triosphate fragment all be it at reduced rates. The 5 mM concentrations used would be in the domain where the concentration effect on binding would be at its highest. Notwithstanding this data it is conceivable that purine analogues deuterated at the C8 position will have enhanced inhibition properties over their non-deuterated counterparts. The role of C8H in GTP hydrolyzing enzymes and the extent to which the C8H also becomes labile during catalysis in GTP has not been defined; however, it is probable that similar mechanisms occur within these enzymes.

In an investigation on the conformational dynamics of *M. tuberculosis *shikimate kinase, the phosphate binding domain was found to be rigid and protected from solvent access, while the shikimate binding domain is highly flexible and the nucleotide binding domain is rigid at low temperatures, becoming more flexible at temperatures above 30°C [55]. The phosphate binding domain contains residues associated with the C8H of ATP [29,55]. It is conceivable that the hydrogen bonding interaction that exists between the Thr17-OH on the phosphate loop, the C8H of ATP and the oxygen on the ATP α-phosphate plays a significant role in the labile nature of the C8H. The fact that the phosphate loop was also found to be rigid could also be significant in the role of the residues in facilitating binding and catalysis associated with the C8H-ATP.

## Methods

### Enzyme source and protein expression and purification

Hexokinase from *Saccharomyces cerevisiae *Type F-300 (Sigma, H4502), Fructose Phosphokinase (Sigma, F0137) and Acetate kinase from *E. coli *(Sigma, A7437) were purchased. The *Mycobacterium tuberculosis *shikimate kinase gene in pET15b (Novagen) was obtained from the group of Chris Abell, Cambridge University, UK. The his-tagged MtSK was produced in *E. coli *BL21(DE3) and purified using the Bio-Rad Profinia Purification System and purity of the enzyme was judged to be 90-95%. The pure protein was dialysed against 50 mM Tris (pH 7.5) and 1,000 mM NaCl. Adenylylated (GS_12_) and deadenylylated (GS_0_) glutamine synthetase were prepared as outlined below.

### Production of *glnD *and *glnE *Knockout Strains

Knockout strains for the production of fully adenylylated (*glnD *knockout) or fully deadenylylated GS (*glnE *kockout) were made from the *E. coli *YMC11 using the Quick & Easy *E. coli *Gene Deletion Kit (Gene Bridges GmbH), designed to knockout or alter genes on the *E. coli *chromosome. Red/ET recombination allows the exchange of genetic information in a base pair precise and specific manner. An FRT-flanked kanamycin resistance marker cassette is supplied with the kit which can be used to replace a gene on the *E. coli *chromosome. The use of a FRT-flanked resistance cassette for the replacement of the targeted gene allows the subsequent removal of the selection marker by a FLP-recombinase step, involving the transformation of an FLP-expression plasmid into the cells and subsequent expression of an FLP site-specific recombinase. The genes for the recombinant proteins are under the control of an inducible promoter and the plasmid carries a temperature sensitive origin of replication for a convenient removal of the plasmid after recombination. In order to produce fully adenylylated GS, it is necessary to knockout the uridylyltransferase, coded by the *gln*D gene. Primers were designed to the *E. coli gln*D gene. These primers contained a region specific to the *gln*D gene adjoining a sequence specific to the FRT cassette (underlined, see below). In a similar fashion, to produce fully deadenylylated GS, the adenylyltrasnferase, coded by the *gln*E gene, needs to be knocked out. Primers were therefore designed to the *E. coli gln*E gene. These primers contained a region specific to the *gln*E gene adjoining a sequence specific to the FRT cassette (underlined, see below). Both knockout strains were produced using the primers as described in the kit protocol. The only deviation from the protocol, was that *Bam*HI restriction sites were incorporated in the ends of the primers (shown in bold). This enabled the PCR product to be cloned into pGEM T-Easy (Promega Corporation), and then cut out of the pGEM construct as a *Bam*HI fragment. This facilitated production of the cassette in sufficient quantity for the transformation step, as it was found to be extremely difficult to produce enough of the cassette by PCR alone. Once integration of the cassette was confirmed by selection on kanamycin plates, a PCR product was produced using primers designed to the sequence of the *gln*D or *gln*E gene, either side of the integration site. This PCR product was then sequenced to confirm integration. The kanamycin resistance marker was removed using the 706-FLP plasmid carrying the site-specific recombinase. The removal of the marker was also confirmed by sequencing, as above. Primers used to create *glnD *and *glnE *knockout strains of *E. coli *YMC11 were:

*glnD *sense primer,

5'-ga**ggatcc**cagaaccagcgccatcagcgttaccatggcaccagctacaaccttgaaccaattaaccctcactaaagggcg-3'; *glnD *antisense primer,

5'-gt**ggatcc**gcgatatcgtgaaacagcgcggcgatgaaaatcagctcagttgacggcagtaatacgactcactatagggctc-3'; *glnE *sense primer,

5'-ga**ggatcc**tgcgcctgtttgaactgacgcagcgcctcaagctgttgctcttcgtcatcaattaaccctcactaaagggcg-3'; *glnE *antisense primer,

5'-gt**ggatcc**aggtgttccagctcattcgcggcggacgcgaaccgtcgctgcaatcgcgctaatacgactcactatagggctc-3'.

### Purification of *E. coli *glutamine synthetase

GS_12 _and GS_0 _were purified from recombinant *E. coli *YMC11 *glnD *and *glnE *knockout strains. *E. coli *YMC11 *glnD*^- ^strain producing GS_12 _and the *E. coli *YMC11 *glnE*^- ^strain producing GS_0_. The culturing protocols used were as outlined in the Additional Files. The enzyme concentration and purity were determined by Quant - IT™ Protein Assay Kit (Invitrogen, USA) and the purity assessed by SDS-PAGE [56]. The purity of the enzyme was judged to be 90-95%.

### C8-D ATP synthesis

The synthesis ATP and ADP deuterated at the C8 position (C8-D ATP and C8-D ADP) was carried out based on the method of [50]. A 20 mM solution of Na_2_ATP in D_2_O containing 60 mM triethylamine (TEA) was incubated at 60ºC for 144 hours. The TEA was removed by twice passing the solution over a Dowex 20W ion-exchange resin in the acid form. The pH of the solution was adjusted to pH 12 with NaOH prior to the second pass over the resin. The pH of the solution was adjusted to pH 6.3 prior to freeze drying. The extent of the deuteration of the C8 proton was determined by ^1^H NMR and mass spectroscopy (Additional Files 1, Figure S5A & S5B). The ^1^H NMR was carried out on a Varian VNMRS 600 MHz NMR in D_2_O.

### Steady-State Kinetic Analysis

The shikimate kinase assay contained: 100 mM potassium phosphate buffer (pH 6.8), 500 mM KCl, 10 nM enzyme, and varying amounts of ATP, shikimic acid and MgCl_2_. These were kept at a constant ratio of 1:1:2 for ATP: MgCl_2_: shikimic acid. The ATP concentrations ranged between 0.2 and 10 mM. The final volume was 100 μl, and the reaction was incubated at 37°C for 20 minutes, before being terminated by the addition of 5 μl 200 mM EDTA. The production of ADP was analysed by HPLC. The hexokinase assay contained; 100 mM Phosphate buffer pH 6.8, 10 mM D-Glucose, 250 mM KCl, MgCl_2 _and ATP was kept at a 1:1 ratio at concentration between 0.2 mM-3 mM. Hexokinase was added to a final concentration of 0.0002 U/ml. The assay was incubated at 37°C for 15 minutes and stopped by the addition of 1 μl of 50% TCA. The formation of ADP was analysed by HPLC. The acetate kinase assay contained; 100 mM Phosphate buffer pH 6.8, 10 mM Sodium Acetate, 250 mM KCl, MgCl_2 _and ATP was kept at a 1:1 ratio at concentration between 0.2 mM-3 mM. The assay was incubated at 30°C for 30 minutes and stopped by the addition of 1 μl of 50% TCA. Acetate kinase was added to a final concentration of 0.0004 U/ml. The formation of ADP was analysed by HPLC. The phosphofructokinase assay contained; 100 mM Phosphate buffer pH 6.8, 10 mM Fructose-6-Phosphate, 250 mM KCl, MgCl_2 _and ATP was kept at a 1:1 ratio at concentration between 0.2 mM-3 mM. The assay was incubated at 37°C for 15-30 minutes and stopped by the addition of 1 μl of 50% TCA. The formation of ADP was analysed by HPLC. The effect of the concentration of ATP and C8D-ATP on the specific activity of GS_12_, and GS_0 _was determined at concentrations ranging from 150 to 3000 M ATP and C8D-ATP in assays containing 4 mM Na-glutamate, 4 mM NH_4_Cl, 5.4 mM NaHCO_3 _in 20 mM imidazole buffer. The GS_0 _assay was carried out at pH 7.4 (± pH 0.05), and at MgCl_2 _concentrations equivalent to 3 times the ATP concentration. The GS_12 _assay was carried out at pH 6.6 (± pH 0.05), and at MnCl_2 _concentrations equivalent to 3 times the ATP concentration. The reaction was stopped by the addition of tri-chloroacetic acid to give a pH of 2-3.

The assay solutions were centrifuged prior to HPLC analysis. The assays for adenosine, AMP, ADP ATP were carried out using Phenomenex 5 μ LUNA C_18 _column with the mobile phase containing PIC A (Waters Coorporation), 250 ml acetonitrile, 7 g KH_2_PO_4 _per litre water. The flow rate of the mobile phase was 1 ml/minute with UV detection. All specific enzyme activities were expressed as moles ADP formed per minute per milligram protein.

The selectivity of a number of kinases for C8D-ATP was determined by carrying out the enzyme activity in the presence ATP, C8D-ATP and assays containing ATP and C8D-ATP at in a 1:1 ratio equivalent to the total concentration used in the ATP and C8D-ATP assays.

## Authors' contributions

CPK defined the concept and experiments of this study. RLR, AS and LCO cloned expressed and purified GS in both forms. RLR, AS, TCN and CPK performed assays and collected data for the various enzyme assays. CPK and TCN produced C8D-ATP. Quality control by MS and NMR on C8D-ATP was carried out by CPK and PAS. High resolution MS carried out by PAS. CPK and RLR drafted the manuscript, where other authors helped proof-read the manuscript. All authors have read and approved the final manuscript.

## Supplementary Material

Additional file 1 **General**.
The selectivity of a number of kinases for C8D-ATP was assessed by determining the steady state enzyme activity in the presence ATP, C8D-ATP and assays containing ATP and C8D-ATP at in a 1:1 ratio equivalent to the total concentration used in the ATP and C8D-ATP assays. These assays were run in excess of 10% ATP (C8D-ATP) conversion to endeavour to ensure sufficient ATP had been utilized to see the effect, but not to the point where if C8D-ATP was being preferentially utilized the ATP concentration exceeded the C8D-ATP and this concentration differential then impacted on the data. Using the equation and constants obtained for the non-linear best-fit from the GraphPad Prism software the theoretical conversion of ATP and C8D-ATP was estimated in the assays containing ATP and C8D-ATP at in a 1:1.
Analytical Methods
General. All chemicals for UPLC-MS work were of ultra-pure LC-MS grade and purchased from Fluka (Steinheim, Germany) while ultra-pure solvents were purchase from Honeywell (Burdick & Jackson, Muskegon, USA). Ultra-pure water was generated from a Millipore Elix 5 RO system and Millipore Advantage Milli-Q system (Millipore SAS, Molsheim, France).
**Instrumental**. A Waters UPLC coupled in tandem to a Waters SYNAPT G1 HDMS mass spectrometer was used to generate accurate mass data. Chromatographic separation was done utilising a Waters HSS T3 column (150 mm × 2.1 mm, 1.8 μm) thermostatted at 60°C. A binary solvent mixture was used consisting of water (Eluent A) containing 10 mM ammonium acetate (natural pH of 6.8) and acetonitrile (Eluent B). The initial conditions were 100% A for two minutes followed by a linear gradient to 5% A: 95% B at six minutes. The column was allowed to wash for one minute where after the system was re-equilibrated using the initial conditions. The runtime was 10 minutes and the injection volume was 10 μL.
The SYNAPT G1 mass spectrometer was used in V-optics and operated in electrospray mode. Leucine enkephalin (50 pg/mL) was used as reference calibrant to obtain typical mass accuracies between 1 and 3 mDa. The mass spectrometer was operated in positive mode with a capillary voltage of 2.5 kV, the sampling cone at 30 V and the extraction cone at 4 V. The source temperature was 120°C and the desolvation temperature was set at 350°C. Nitrogen gas was used as the nebulisation gas at a flow rate of 450 L/h. The software used to control the hyphenated system and do all data manipulation was MassLynx 4.1 (SCN 704).
Additional figures figures.
Figure S1A. Shikimate kinase steady state assays run to 3-5% conversion. Mass spectra showing the relative in intensities for ATP and C8D-ATP from assays run using 1.8 mM ATP, 1.8 mM C8D-ATP and 0.9 mM ATP:0.9 mM C8D-ATP.
Figure S1B. Shikimate kinase steady state assays run to ≈ 0% conversion. Mass spectra showing the relative in intensities for ATP and C8D-ATP from an assay run using 0.06 mM ATP:0.06 mM C8D-ATP and 1.5 mM ATP:1.7 mM C8D-ATP.
Figure S2A. Hexokinase steady state assays run to 3-5% conversion. Mass spectra showing the relative in intensities for ATP and C8D-ATP from assays run using 0.5 mM ATP, 0.5 mM C8D-ATP and 0.25 mM ATP:0.25 mM C8D-ATP.
Figure S2. Hexokinase steady state assays run to ≈10% conversion. Mass spectra showing the relative in intensities for ATP and C8D-ATP from assays run using 0.2 mM ATP:0.2 mM C8D-ATP and 1.5 mM ATP:1.5 mM C8D-ATP.
Figure S3A. Acetate kinase steady state assays run to 3-5% conversion. Mass spectra showing the relative in intensities for ATP and C8D-ATP from assays run using 0.5 mM ATP, 0.5 mM C8D-ATP and 0.25 mM ATP:0.25 mM C8D-ATP.
Figure S3B. Acetate kinase steady state assays run to ≈5% conversion. Mass spectra showing the relative in intensities for ATP and C8D-ATP from assays run using 0.125 mM ATP:0.125 mM C8D-ATP and 1.25 mM ATP:1.25 mM C8D-ATP.
Figure S4A. Phosphofructokinase steady state assays run to 3-5% conversion. Mass spectra showing the relative in intensities for ATP and C8D-ATP from assays run using 0.7 mM ATP, 0.7 mM C8D-ATP and 0.35 mM ATP:0.35 mM C8D-ATP.
Figure S4B. Phosphofructokinase steady state assays run to ≈5% conversion. Mass spectra showing the relative in intensities for ATP and C8D-ATP from assays run using 0.15 mM ATP:0.15 mM C8D-ATP.
Figure S5A. ^1^H NMR spectrum of C8-D ATP showing the deuteration of the C8 position.
Figure S5B. Mass spectroscopy analysis of ATP
Table S1A - Comparison of the kinetic constants obtained, in defining the effect of the concentration of ATP and C8D-ATP, by comparing the kinetic models using GraphPad Prism software.
Table S1B - Comparison of the kinetic constants obtained, in defining the effect of the concentration of ATP and C8D-ATP, by comparing the kinetic models using GraphPad Prism software as expressed by the 95% confidence intervals. Click here for file
