# Supplementary material for: The role of the C8 proton of ATP in the regulation of phosphoryl transfer within kinases and synthetases
Source: BMC Biochem. 2011 Jul 13;12:36. doi: 10.1186/1471-2091-12-36 (PMC3145573; doi:10.1186/1471-2091-12-36)
Supplement: Additional file 1 — General. The selectivity of a number of kinases for C8D-ATP was assessed by determining the steady state enzyme activity in the presence ATP, C8D-ATP and assays containing ATP and C8D-ATP at in a 1:1 ratio equivalent to the total concentration used in the ATP and C8D-ATP assays. These assays were run in excess of 10% ATP (C8D-ATP) conversion to endeavour to ensure sufficient ATP had been utilized to see the effect, but not to the point where if C8D-ATP was being preferentially utilized the ATP concentration exceeded the C8D-ATP and this concentration differential then impacted on the data. Using the equation and constants obtained for the non-linear best-fit from the GraphPad Prism software the theoretical conversion of ATP and C8D-ATP was estimated in the assays containing ATP and C8D-ATP at in a 1:1. Analytical Methods General. All chemicals for UPLC-MS work were of ultra-pure LC-MS grade and purchased from Fluka (Steinheim, Germany) while ultra-pure solvents were purchase from Honeywell (Burdick & Jackson, Muskegon, USA). Ultra-pure water was generated from a Millipore Elix 5 RO system and Millipore Advantage Milli-Q system (Millipore SAS, Molsheim, France). Instrumental. A Waters UPLC coupled in tandem to a Waters SYNAPT G1 HDMS mass spectrometer was used to generate accurate mass data. Chromatographic separation was done utilising a Waters HSS T3 column (150 mm × 2.1 mm, 1.8 μm) thermostatted at 60°C. A binary solvent mixture was used consisting of water (Eluent A) containing 10 mM ammonium acetate (natural pH of 6.8) and acetonitrile (Eluent B). The initial conditions were 100% A for two minutes followed by a linear gradient to 5% A: 95% B at six minutes. The column was allowed to wash for one minute where after the system was re-equilibrated using the initial conditions. The runtime was 10 minutes and the injection volume was 10 μL. The SYNAPT G1 mass spectrometer was used in V-optics and operated in electrospray mode. Leucine enkephalin (50 pg/mL) was used a [file 1471-2091-12-36-S1.PDF]

Additional Files 1

Figure S1A.Shikimate kinase: (≈ 3-5% substrate conversion)

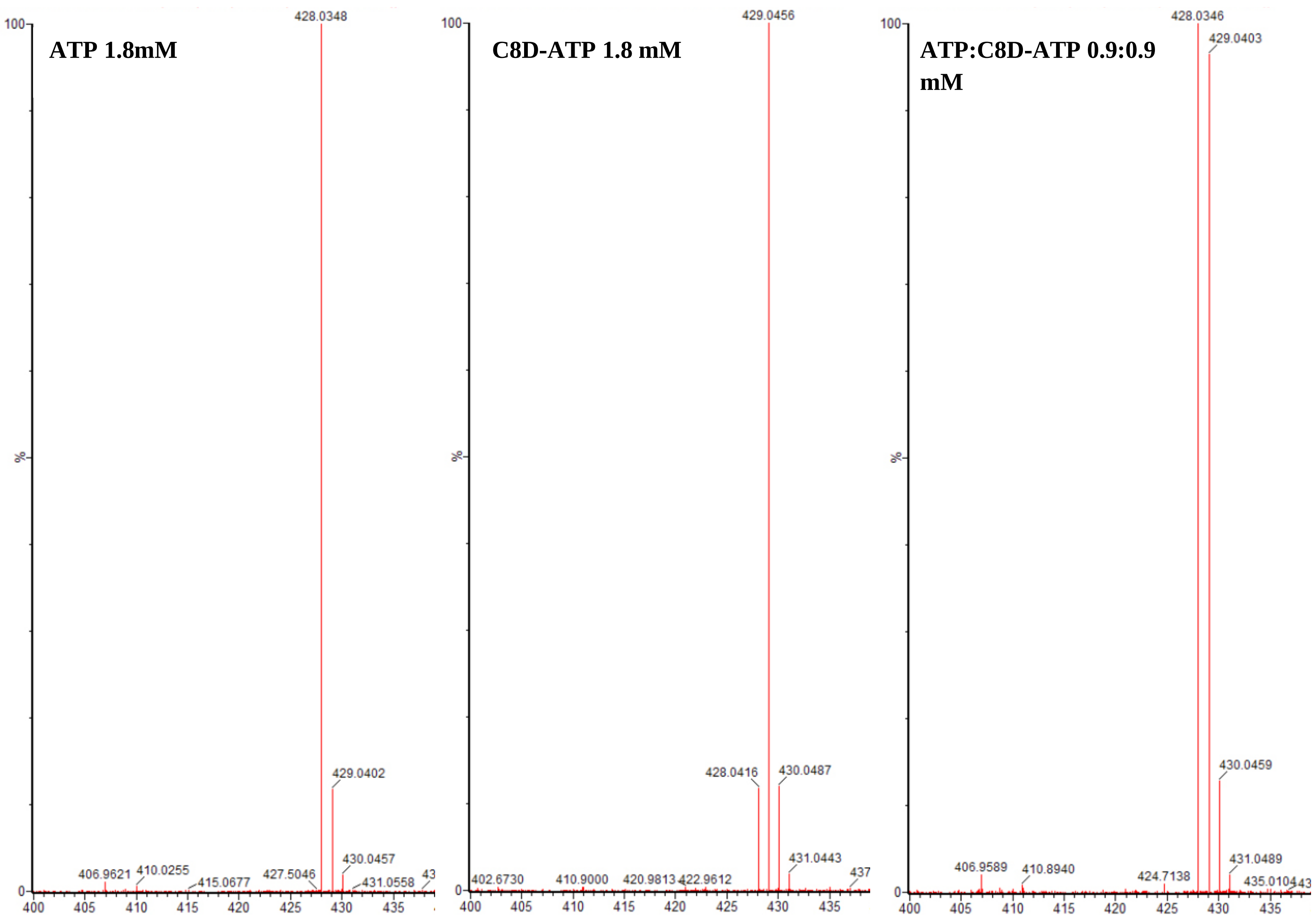

Figure S1B. Shikimate kinase: ATP:C8D-ATP 1:1 ratio (≈ 5-10% substrate conversion)

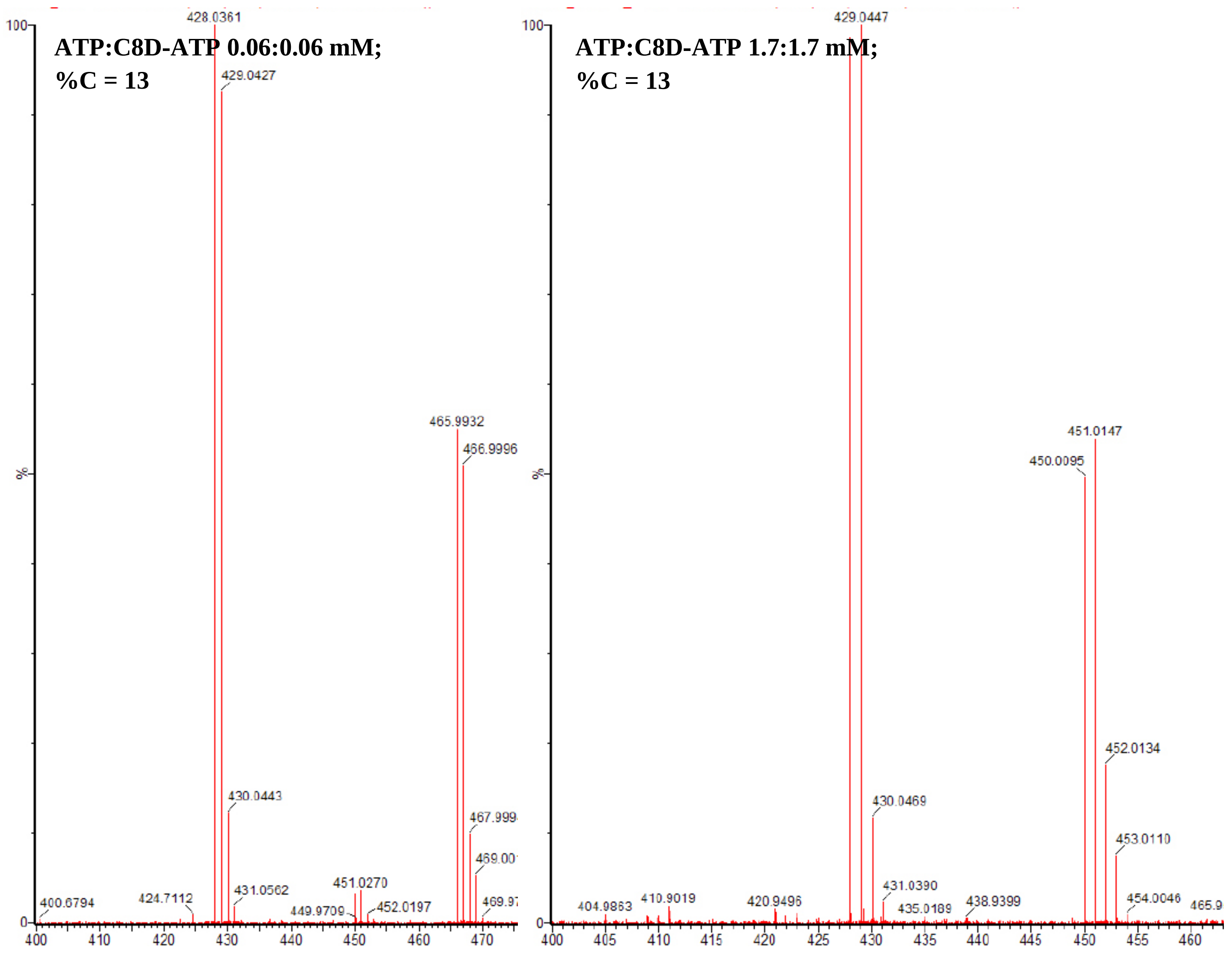

Figure S2A. Hexokinase: (≈ 3-5% substrate conversion)

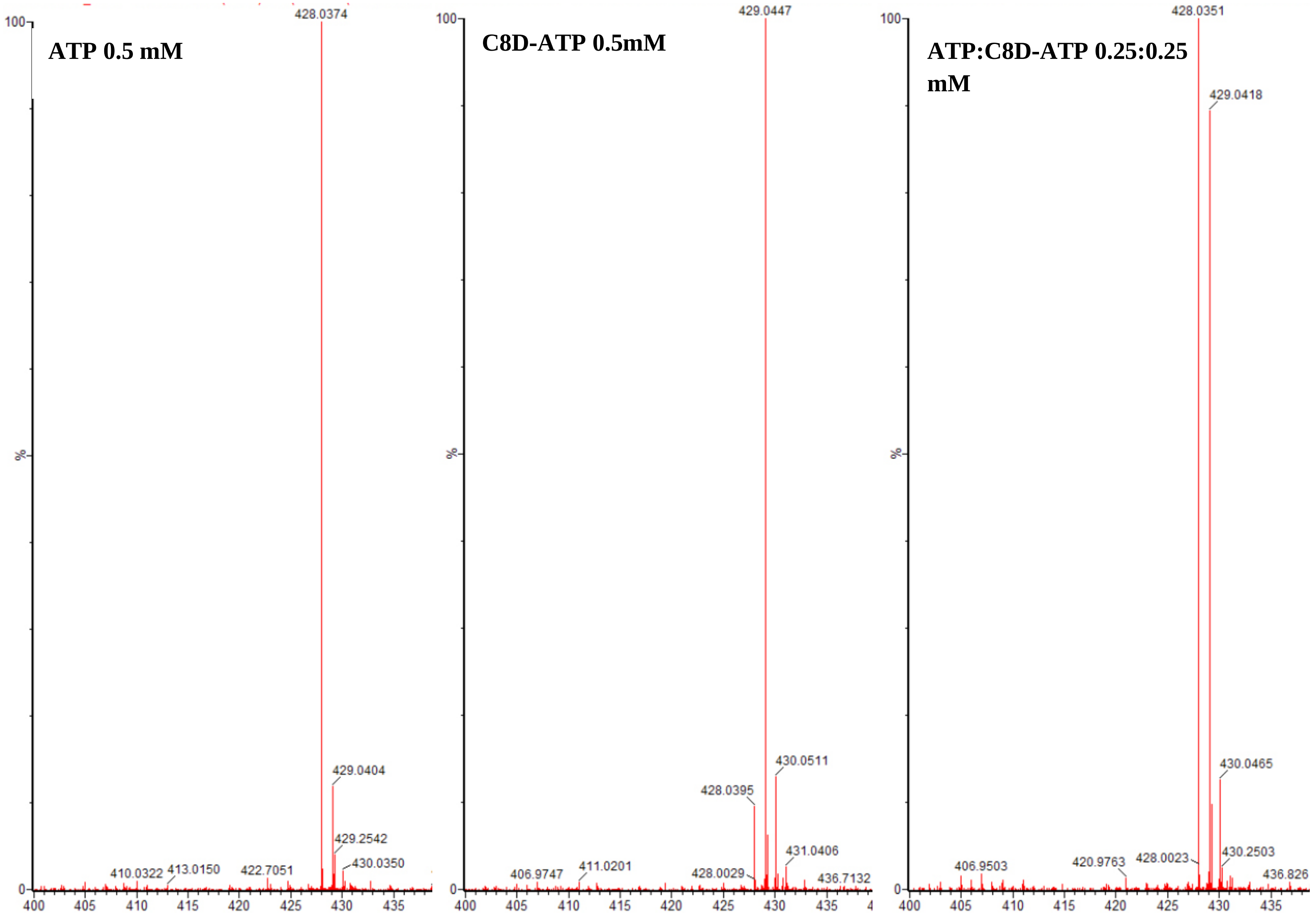

Figure S2B. Hexokinase: ATP:C8D-ATP 1:1 ratio (≈ 10-12% substrate conversion)

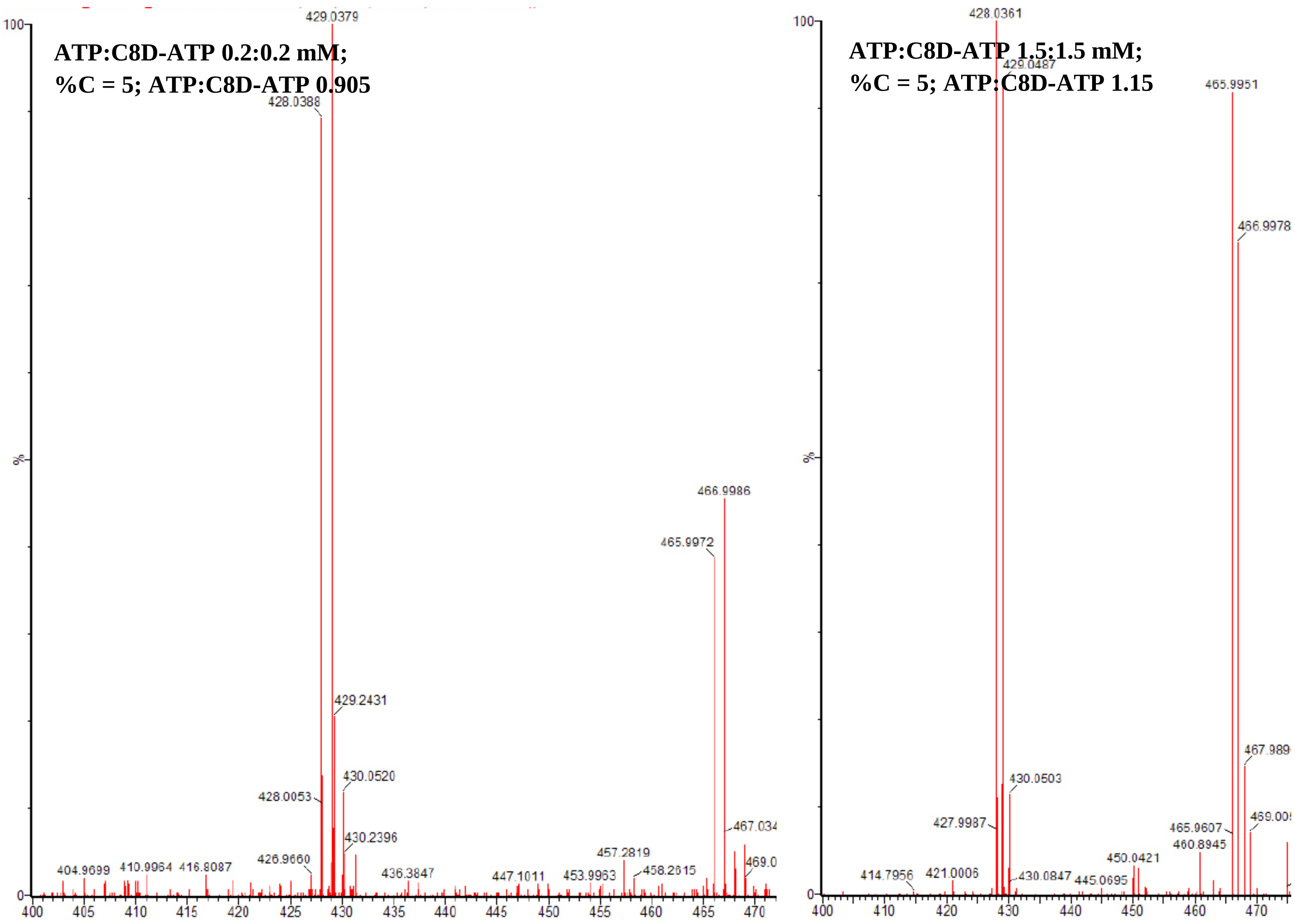

Figure S3A. Acetate Kinase: (≈ 3-5% substrate conversion)

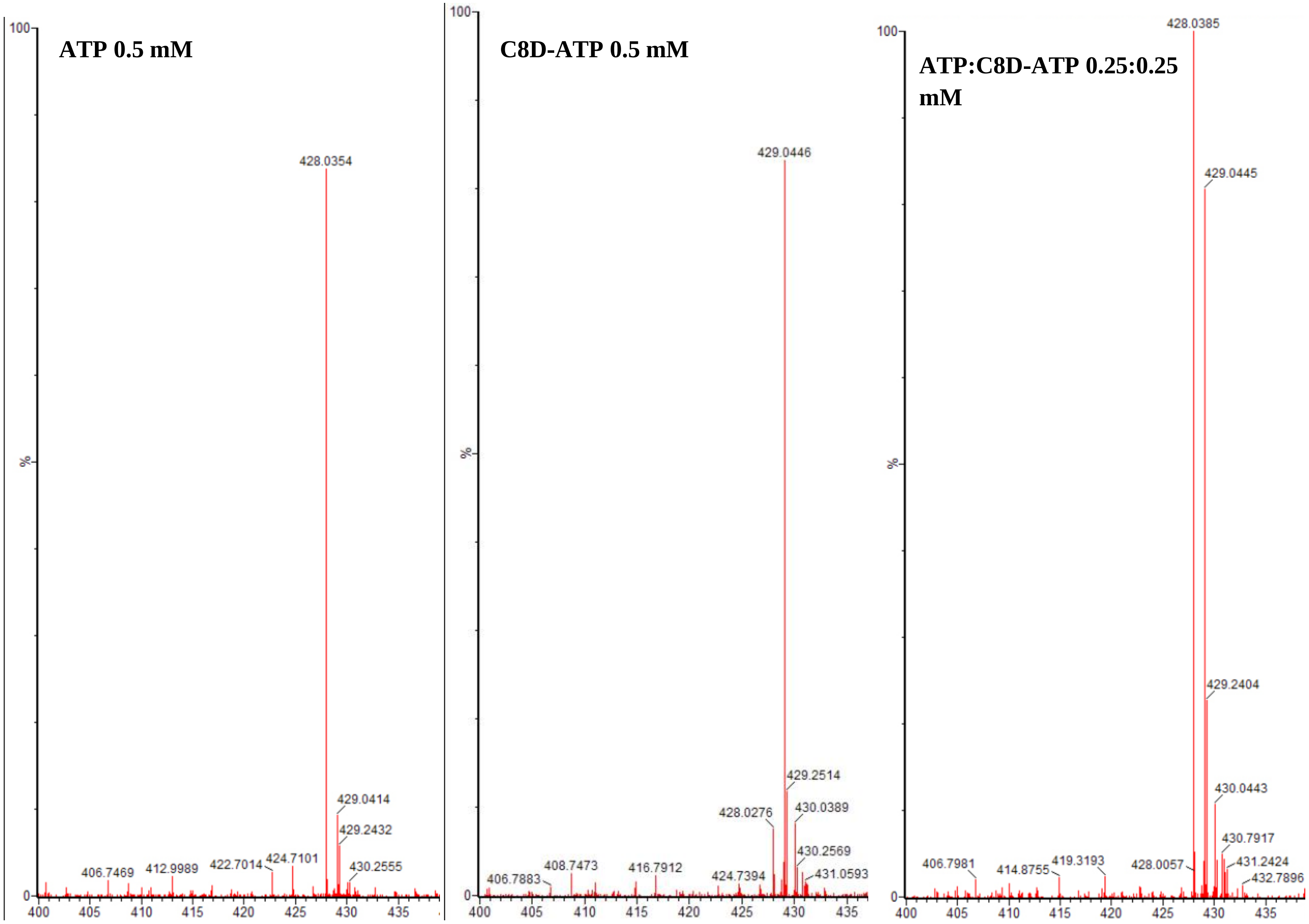

Figure 3A. Acetate Kinase: ATP:C8D-ATP 1:1 ratio (≈ 5% substrate conversion)

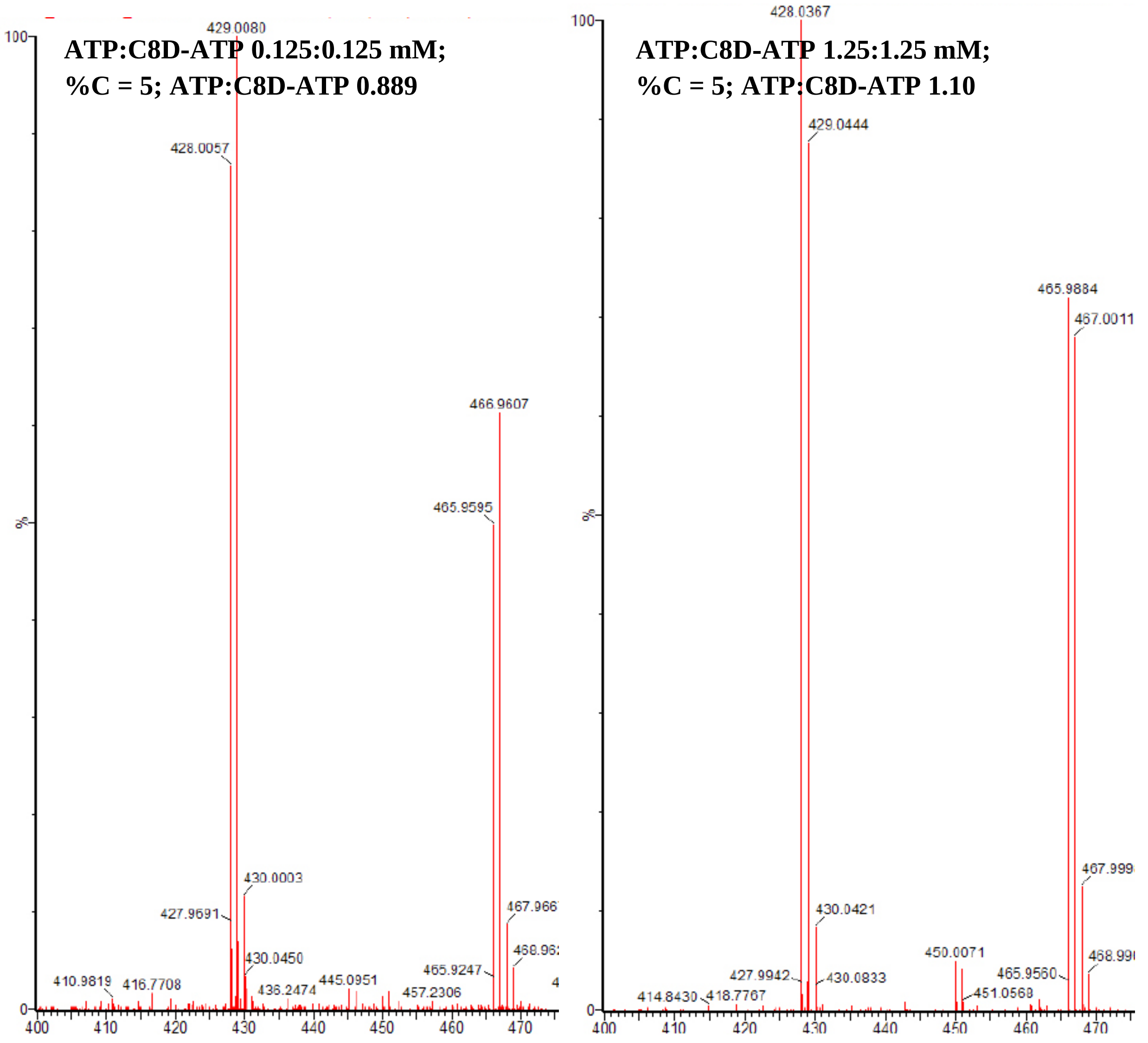

Figure S4A. Phosphofructokinase: (≈ 3-5% substrate conversion)

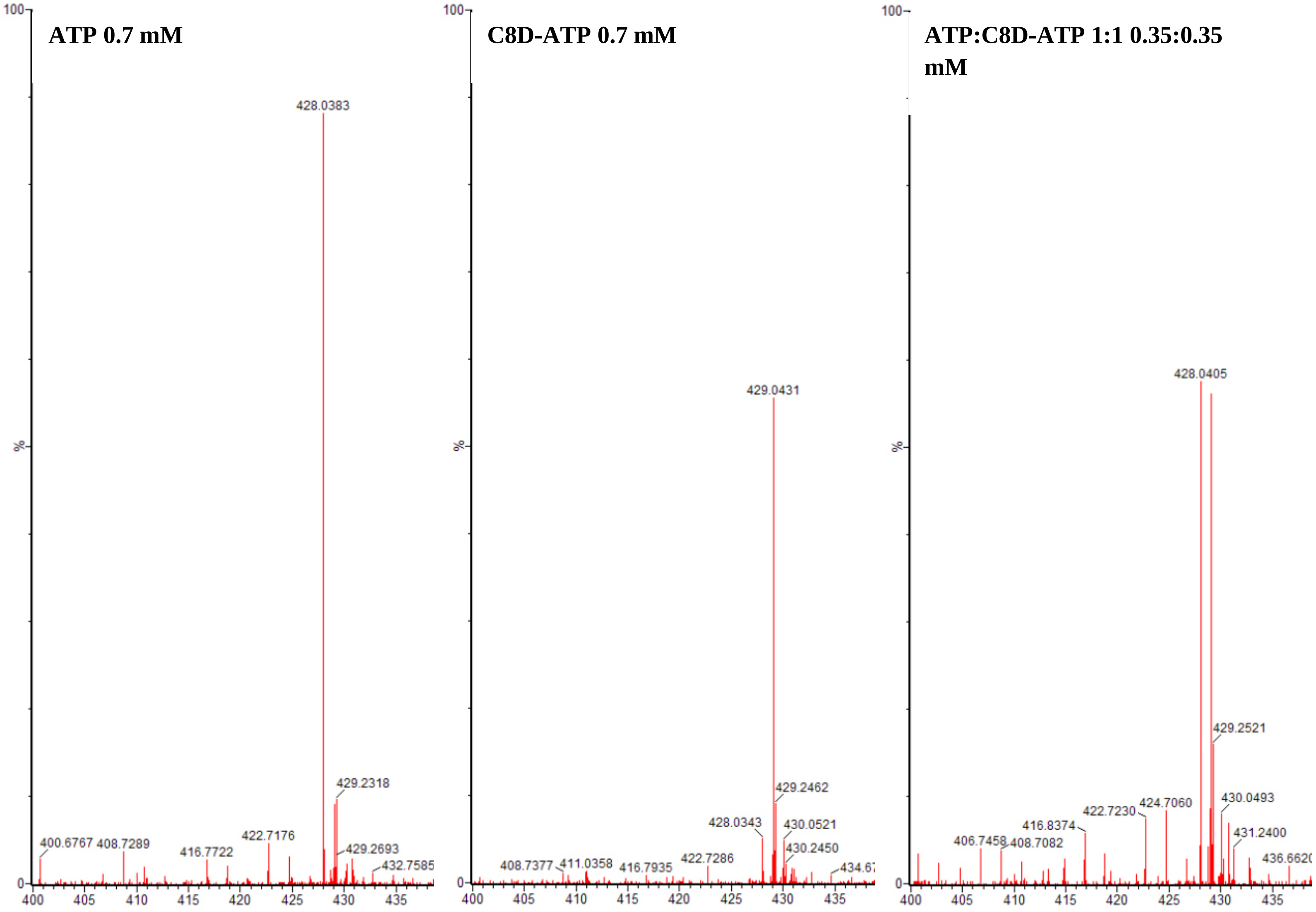

Figure S4B. Phosphofructokinase: ATP:C8D-ATP 1:1 ratio (≈ 9-30% substrate conversion)

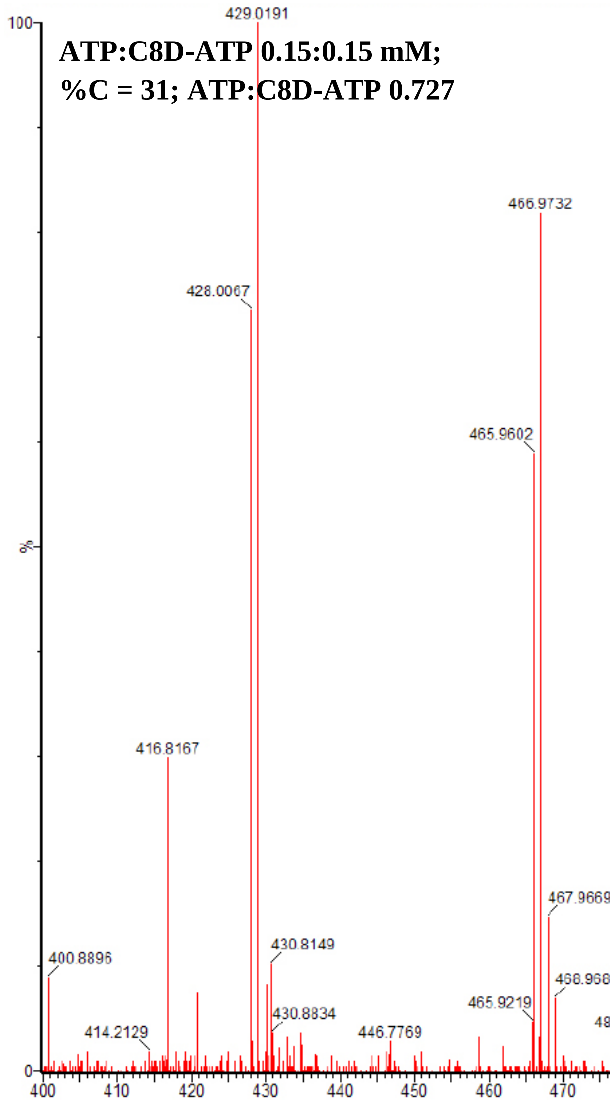

Table S1A – Comparison of the kinetic constants obtained, in defining the effect of the concentration of ATP and C8D-ATP, by comparing the kinetic models using GraphPad Prism software. [a] Root mean square deviation of the data defining the kinetic model. [b] Phosphofructokinase

**Enzyme**	**ATP : Kinetic model**	**C8D-ATP: Kinetic model**	**Enzyme**	**ATP : Kinetic model**	**C8D-ATP: Kinetic model**
**Shikimate kinase**	**Michaelis-Menton**	**Michaelis-Menton**	**Deadenylylated GS**	**Allosteric sigmoidal**	**Allosteric sigmoidal**
$\boldsymbol{v_{max}}$	**2339±600**	**2433±600**	$\boldsymbol{v_{max}}$	**11.49±1.31**	**13.20±0.651**
$\boldsymbol{K_m}$ **or** $\boldsymbol{K'}$	**0.0065±0.003**	**0.0128±0.007**	$\boldsymbol{K_m}$ **or** $\boldsymbol{K'}$	**30.88±4.44**	**6.774±2.331**
**RMSD[a]**	**0.9543**	**0.9866**	**h**	**3.108±0.316**	**4.077±0.787**
**Shikimate kinase**	Allosteric sigmoidal	Allosteric sigmoidal	**RMSD[a]**	**0.9972**	**0.9827**
$\boldsymbol{v_{max}}$	1850±865	1631±604	Deadenylylated GS	Michaelis-Menton	Michaelis-Menton
$\boldsymbol{K_m}$ **or** $\boldsymbol{K'}$	0.0057±0.003	0.010±0.003	v_{max}	Fit ambiguous	51.29±36.46
**H**	1.229±0.612	1.256±0.348	K_m or K'	Fit ambiguous	10.21±9.291
**RMSD[a]**	0.9543	0.9866	h	Fit ambiguous	
**Hexokinase**	**Allosteric sigmoidal**	**Michaelis-Menton**	RMSD[a]	Fit ambiguous	0.9827
$\boldsymbol{v_{max}}$	**1226±189**	**2004 ± 289.9**	**PFK[b]**	**Allosteric sigmoidal**	**Allosteric sigmoidal**
$\boldsymbol{K_m}$ **or** $\boldsymbol{K'}$	**3.263±0.662**	**4.737 ± 0.9864**	$\boldsymbol{v_{max}}$	**111.3±10.16**	**45.18±1.012**
**H**	**1.759±0.207**		$\boldsymbol{K_m}$ **or** $\boldsymbol{K'}$	**2.265±0.320**	**0.097±0.031**
**RMSD[a]**	**0.9959**	**0.9919**	**h**	**1.371±0.098**	**1.785±0.208**
**Hexokinase**	Michaelis-Menton	Allosteric sigmoidal	**RMSD[a]**	**0.9982**	**0.9248**
$\boldsymbol{v_{max}}$	Fit ambiguous	1893±940.7	PFK[b]	Michaelis-Menton	Michaelis-Menton
$\boldsymbol{K_m}$ **or** $\boldsymbol{K'}$	Fit ambiguous	4.419±2.749	v_{max}	202.9±29.80	51.72±2.339
**H**	Fit ambiguous	1.020	K_m or K'	4.972±1.029	0.3188±0.06178
**RMSD[a]**	Fit ambiguous	0.9919	RMSD[a]	0.9932	0.9439
**Acetate kinase**	**Allosteric sigmoidal**	**Michaelis-Menton**	**Adenylylated GS**	**Allosteric sigmoidal**	**Allosteric sigmoidal**
$\boldsymbol{v_{max}}$	**22.29±3.046**	**19.13±2.103**	$\boldsymbol{v_{max}}$	**8.241±0.787**	**6.540±0.271**
$\boldsymbol{K_m}$ **or** $\boldsymbol{K'}$	**1.323±0.353**	**1.081±0.289**	$\boldsymbol{K_m}$ **or** $\boldsymbol{K'}$	**15.22±8.776**	**0.603±0.151**
**H**	**1.658±0.302**		**H**	**3.288±**	**3.258±0.589**
**RMSD[a]**	**0.9880**	**0.9704**	**RMSD[a]**	**0.9822**	**0.9924**
**Acetate kinase**	Michaelis-Menton	Allosteric sigmoidal	**Adenylylated GS**	Michaelis-Menton	Michaelis-Menton
$\boldsymbol{v_{max}}$	40.37±7.767	15.23±1.996	v_{max}	Fit ambiguous	Fit ambiguous
$\boldsymbol{K_m}$ **or** $\boldsymbol{K'}$	3.207±0.9510	1.412±0.3615	K_m or K'	Fit ambiguous	Fit ambiguous
**H**		0.5990±0.2642	h	Fit ambiguous	Fit ambiguous
**RMSD[a]**	0.9774	0.9796	RMSD[a]	Fit ambiguous	Fit ambiguous

Table S1B - Comparison of the kinetic constants obtained, in defining the effect of the concentration of ATP and C8D-ATP, by comparing the kinetic models using GraphPad Prism software as expressed by the 95% confidence intervals.

**Enzyme**	**ATP : Kinetic model**	**C8D-ATP: Kinetic model**	**Enzyme**	**ATP : Kinetic model**	**C8D-ATP: Kinetic model**
**Shikimate kinase**	**Michaelis-Menton**	**Michaelis-Menton**	**Deadenylylated GS**	**Allosteric sigmoidal**	**Allosteric sigmoidal**
$\boldsymbol{v_{max}}$	**854 to 3824**	**1.060 to 3.806**	$\boldsymbol{v_{max}}$	**8.476 to 14.51**	**11.70 to 14.71**
$\boldsymbol{K_m}$ **or** $\boldsymbol{K'}$	**0.0 to 14.48**	**1.809 to 23.75**	$\boldsymbol{K_m}$ **or** $\boldsymbol{K'}$	**20.64 to 41.13**	**1.400 to 12.15**
**RMSD**[a]	**0.9543**	**0.9866**	**h**	**2.380 to 3.836**	**2.264 to 5.891**
**Shikimate kinase**	Allosteric sigmoidal	Allosteric sigmoidal	**RMSD**[a]	**0.9972**	**0.9827**
$\boldsymbol{v_{max}}$	-373178 to 4.074e+006	77743 to 3.185e+006	Deadenylylated GS	Michaelis-Menton	Michaelis-Menton
$\boldsymbol{K_m}$ **or** $\boldsymbol{K'}$	0.0 to 12.41	3.096 to 17.10	v_{max}	Fit ambiguous	-31.18 to 133.8
**h**	0.0 to 2.802	0.3620 to 2.150	K_m or K'	Fit ambiguous	0.0 to 31.23
**RMSD**[a]	0.9543	0.9866	h	Fit ambiguous	
**Hexokinase**	**Allosteric sigmoidal**	**Michaelis-Menton**	RMSD[a]	Fit ambiguous	0.9827
$\boldsymbol{v_{max}}$	**764.3 to 1688**	**1318 to 2690**	**PFK**[b]	**Allosteric sigmoidal**	**Allosteric sigmoidal**
$\boldsymbol{K_m}$ **or** $\boldsymbol{K'}$	**1.645 to 4.882**	**2.404 to 7.070**	$\boldsymbol{v_{max}}$	**86.39 to 136.1**	**42.71 to 47.66**
**h**	**1.253 to 2.266**		$\boldsymbol{K_m}$ **or** $\boldsymbol{K'}$	**1.481 to 3.049**	**0.02153 to 0.1723**
**RMSD**[a]	**0.9959**	**0.9919**	**h**	**1.130 to 1.611**	**1.275 to 2.295**
**Hexokinase**	Michaelis-Menton	Allosteric sigmoidal	**RMSD**[a]	**0.9982**	**0.9248**
$\boldsymbol{v_{max}}$	Fit ambiguous	-408.5 to 4195	PFK[b]	Michaelis-Menton	Michaelis-Menton
$\boldsymbol{K_m}$ **or** $\boldsymbol{K'}$	Fit ambiguous	0.0 to 11.14	v_{max}	132.5 to 273.4	46.19 to 57.26
**h**	Fit ambiguous	0.5816 to 1.458	K_m or K'	2.539 to 7.405	0.1727 to 0.4650
**RMSD**[a]	Fit ambiguous	0.9919	RMSD[a]	0.9932	0.9439
**Acetate kinase**	**Allosteric sigmoidal**	**Michaelis-Menton**	**Adenylylated GS**	**Allosteric sigmoidal**	**Allosteric sigmoidal**
$\boldsymbol{v_{max}}$	**15.09 to 29.50**	**13.98 to 24.27**	$\boldsymbol{v_{max}}$	**6.216 to 10.27**	**5.678 to 7.403**
$\boldsymbol{K_m}$ **or** $\boldsymbol{K'}$	**0.4886 to 2.158**	**0.3749 to 1.788**	$\boldsymbol{K_m}$ **or** $\boldsymbol{K'}$	**0.0 to 37.78**	**0.1230 to 1.083**
**h**	**0.9427 to 2.373**		**h**	**1.164 to 5.411**	**1.384 to 5.133**
**RMSD**[a]	**0.9880**	**0.9704**	**RMSD**[a]	**0.9822**	**0.9924**
**Acetate kinase**	Michaelis-Menton	Allosteric sigmoidal	**Adenylylated GS**	Michaelis-Menton	Michaelis-Menton
$\boldsymbol{v_{max}}$	22.46 to 58.28	10.10 to 20.36	v_{max}	Fit ambiguous	Fit ambiguous
$\boldsymbol{K_m}$ **or** $\boldsymbol{K'}$	1.014 to 5.400	0.0 to 1.278	K_m or K'	Fit ambiguous	Fit ambiguous
**h**		0.4823 to 2.341	h	Fit ambiguous	Fit ambiguous
**RMSD**[a]	0.9774	0.9796	RMSD[a]	Fit ambiguous	Fit ambiguous

- the “Fit is ambiguous” i.e. the model could not fit the data
- the resolution of the data as expressed by the standard error
- and confidence intervals of the data.
-

Acetate kinase forms part of the acetate and sugar kinase/Hsc70/actin (ASKHA) structural superfamily (PFam Clan: *Actin_ATPase*:CL0108) [6]. The enzyme is a homodimer and monomer interaction plays a role in the regulation of the enzyme activity and ligand binding with the enzyme active sites functioning in a coordinated half-the-sites manner [7-9].

Yeast hexokinase enzymes are structurally well characterised with each subunit of the homodimer comprising two domains and in the open conformation these domains are separated by a cleft containing the sugar binding site [16-22]. Binding of glucose induces a large conformational change in which the two lobes of the subunit rotate relative to each other. The enzymes also exist in a monomer-dimer association-dissociation equilibrium that is influenced by pH, ionic strength and substrates. There are major differences in the glucose binding behaviour of both forms where binding to dimeric P-I shows strong positive cooperativity, whereas in P-II the two sites are equivalent and binding is non-cooperative [23-26].

The SK crystal structures show that SK exists as a monomer with a single ATP binding site and MgADP induces concerted hinged movements of the shikimate binding and LID domains causing the two domains to move towards each other in the presence of this ligand [29].

PFK from *B. stearothermophilus* is a homo-tetramer with each subunit having a molecular weight of 34 000, which undergoes a concerted two-state allosteric transition [30]. PFK belongs to the PFK-like superfamily (Pfam Clan: *PFK*:CL0240) The enzyme from *B. stearothermophilus* (Bs-PFK) shows hyperbolic Michaelis-Menten kinetics with respect to both Fru-6-P and Mg-ATP, but cooperative kinetics in the presence of allosteric inhibitor phosphoenolpyruvate(PEP) [31].

Escherichia coli GS is a large, metalloenzyme (~624 kDa) comprising 12 identical subunits arranged in two face-to-face hexagonal rings [33]. *E. coli* GS belongs to the glutamine synthetase 1-β group of enzymes that are regulated via adenylylation of a single tyrosine residue [34], with each subunit requiring two structurally implicated divalent cations (either Mg^{2+} or Mn^{2+}) for its catalytic activity.

Figure S5A. ^{1}H NMR spectrum of C8-D ATP showing the deuteration of the C8 position.

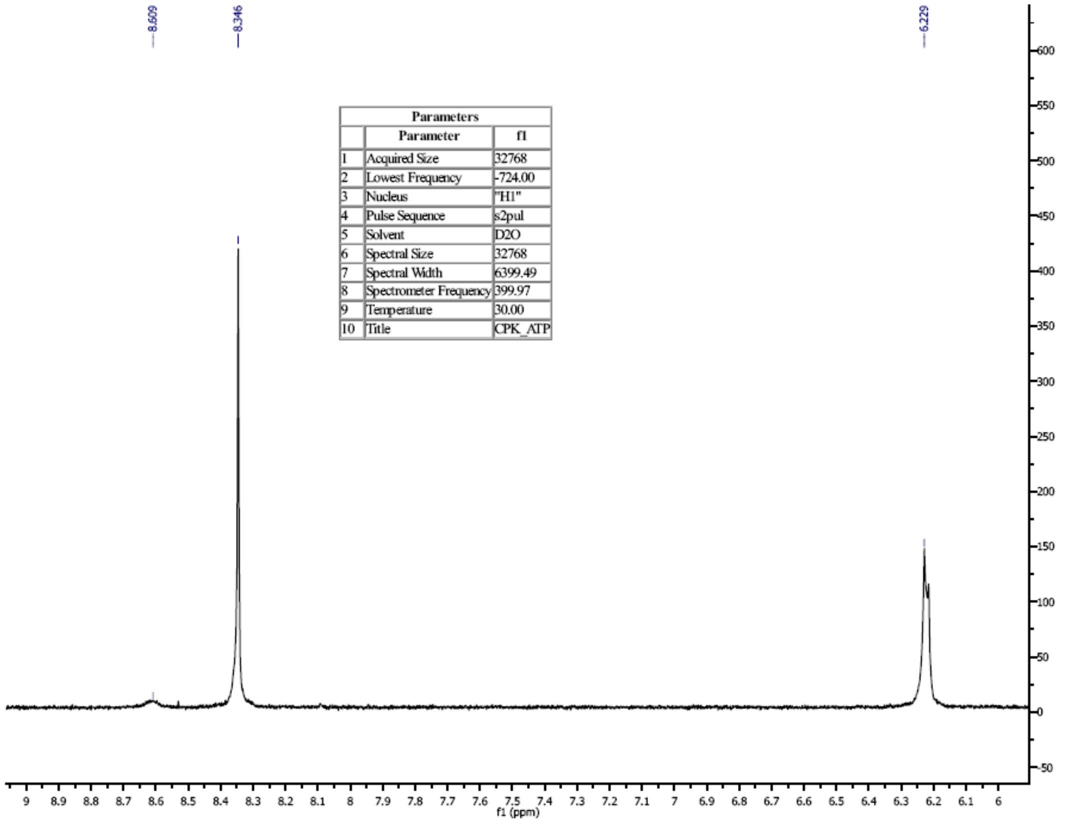

Figure S5B. Mass spectroscopy analysis of ATP

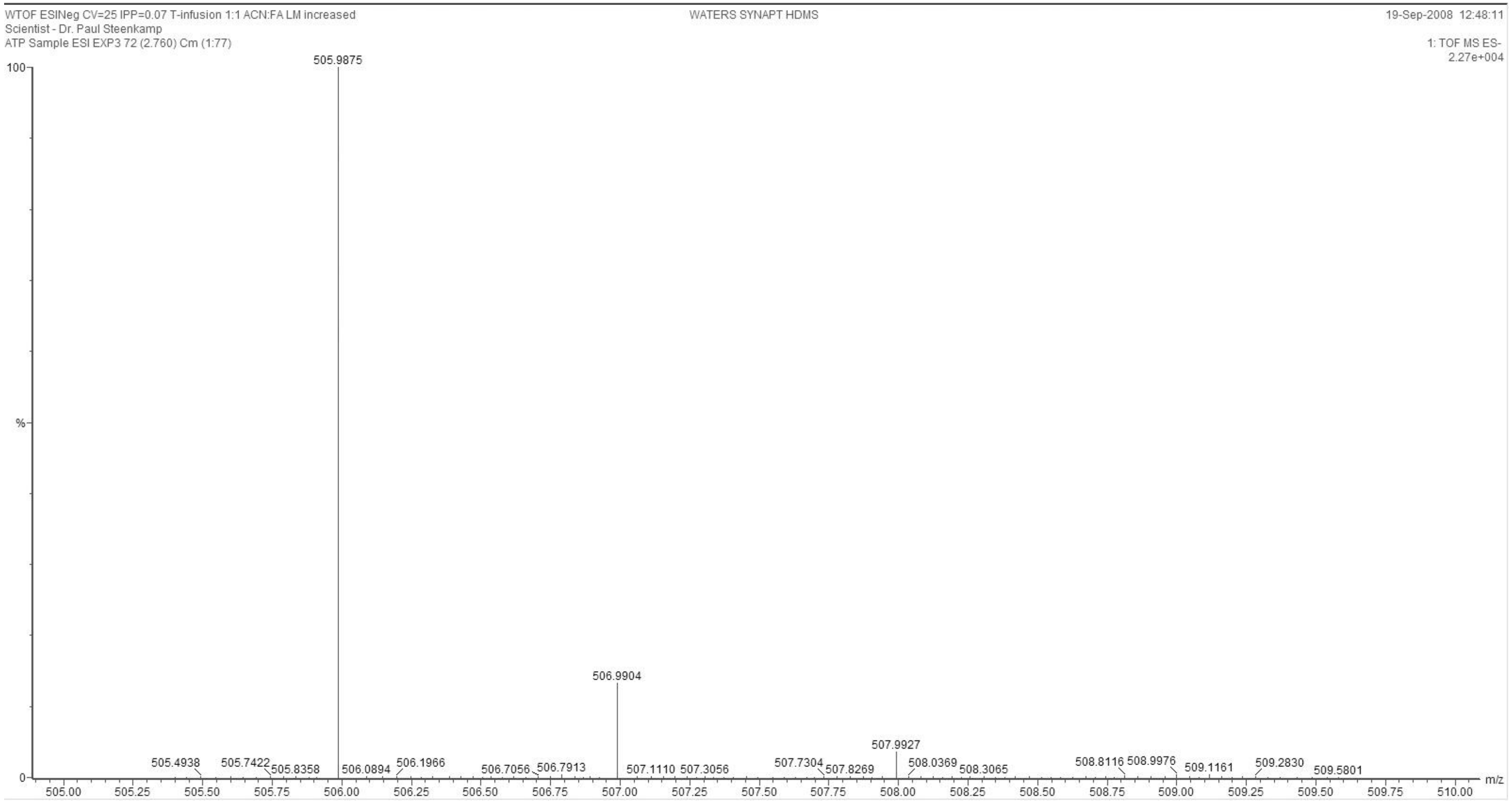

Mass spectroscopy analysis of C8-D ATP

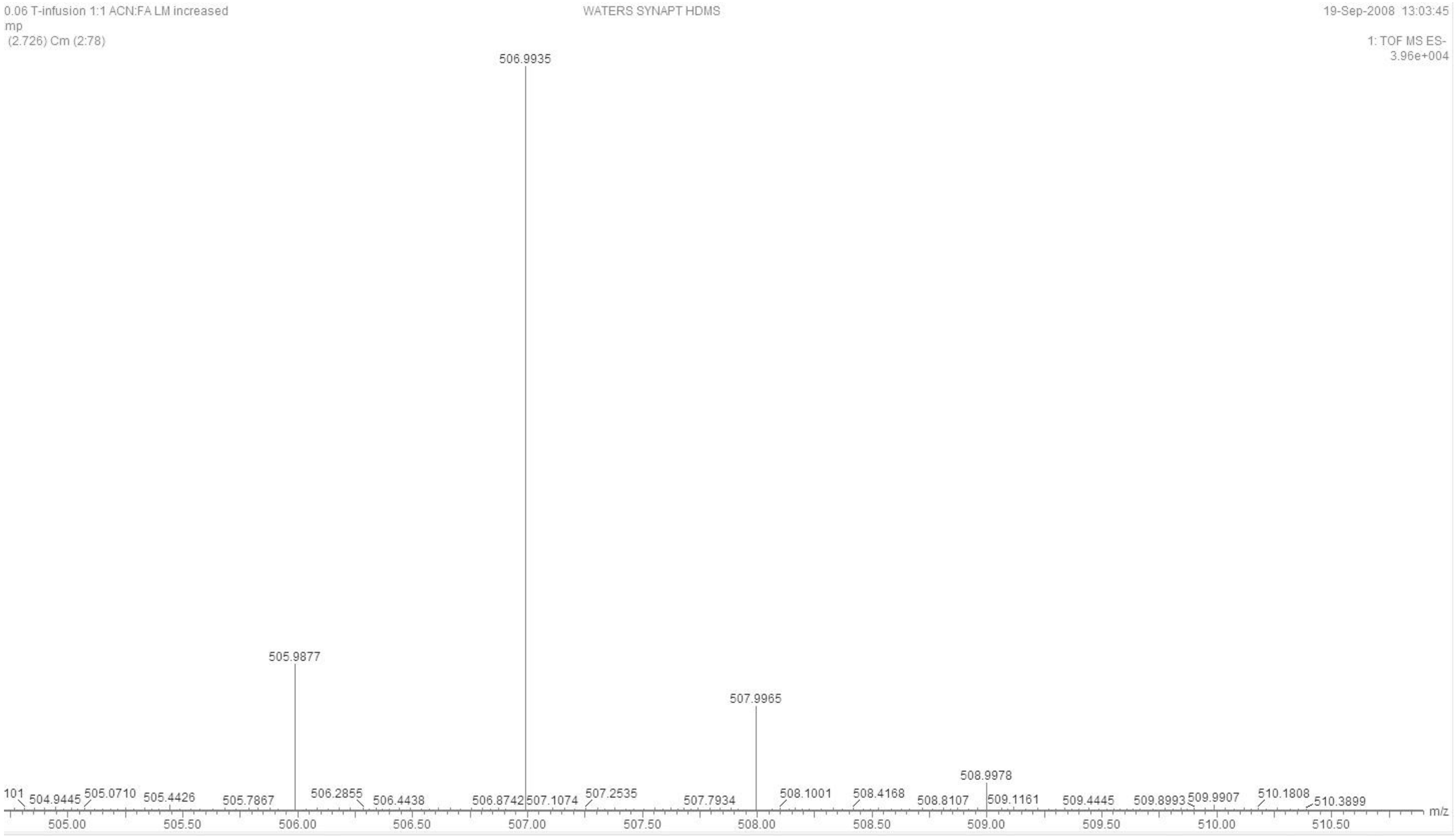

<u>Preparation of GS proteins</u>

Production of Deadenylated *E.coli* GS

<u>Plasmid construct, *E.coli* strain, transformation</u>: The *E.coli* YMC11 (CGSC) strain in which the endogenous GS gene is knocked out was used as the basic expression host. To enhance the expression of fully deadenylylated *E.coli* GS, the endogenous *E.coli* adenylyl transferase gene was also knocked out by homologous recombination to produce the YMC11E strain. A pBSK plasmid vector containing the *glnA* gene encoding *E.coli* GS under the transcriptional control of a constitutive T3 promoter was used to transform YMC11E by electroporation. The resultant recombinant *E.coli* was named pBSK ECgln YMC11E.

<u>*E.coli* Deadenylylated GS expression</u>: pBSK ECgln YMC11E was used to inoculate 50ml LM medium (1% Tryptone, 0.5% yeast extract, 1% NaCl) supplemented with 50 µl Ampicillin (100 µg/ml). The inoculum was grown at 37ºC for 16 hours with shaking at 220 rpm. Subsequently, 1ml of the culture was transferred to 50ml LM medium supplemented with 50 µl AMP_{100}. This second inoculum was again grown at 37ºC for 6 hours with shaking at 220 rpm. Subsequently, 8ml of the culture was transferred to a modified M9 medium (6 g/l Na_2HPO_4, 3 g/l KH_2PO_4, 0.5 g/l NaCl) supplemented with 70mM L-glutamate, Trace salts (4.5mM $CaCl_2.2H_2O$, 6.2mM $FeCl_3.6H_2O$, 0.63mM $ZnCl_2$, 0.64mM $CuSO_4.5H_2O$, 0.76mM $CoCl_2.6H_2O$, 2.4mM $MnCl_2.4H_2O$) 4% Glucose, 1mM $MgSO_4$, 0.1mM $CaCl_2$ and 1mM Thiamine. The culture was grown at 37ºC for 16 hours with shaking at 220 rpm. The cells were harvested from the cultures by centrifugation for 10 min at 16300 x g and the bacterial pellet used for *E.coli* GS purification.

Production of Adenylated *E.coli* GS

Plasmid construct, E.coli strain, transformation: The *E.coli* YMC11 (CGSC) strain lacking the endogenous GS gene was used. However, to enhance the expression of fully adenylated *E.coli* GS, the endogenous *E.coli* uridylyl transferase gene was also knocked out by homologous recombination to produce the YMC11D strain. A pBSK plasmid vector containing the *glnA* gene encoding *E.coli* GS under the transcriptional control of a constitutive T3 promoter was used to transform YMC11D by electroporation. The resultant *E.coli* was named pBSK ECgln YMC11D.

E.coli Adenylylated GS expression: pBSK ECgln YMC11 D was used for the expression of adenylylated GS using a similar method to that described above for deadenylylated GS. The only difference is the addition of 5 mM L-glutamine to the M9 medium.

Purification of Adenylylated and Deadenylylated *E.coli* GS

Step 1 (streptomycin sulphate precipitation): The bacterial pellet was resuspended in 10ml of RBA (10mM Imidazole pH 7, 10mM $MnCl_2$) and sonicated for 20 min on a 50% on/off cycle, followed by centrifugation for 10min at 12100 x g and 4ºC to collect the soluble *E.coli* lysate. A precipitation step was carried out by adding streptomycin sulphate to a final concentration of 1% and stirring at 4ºC for 10 min. After centrifugation for 10 min at 12100 x g and 4ºC the supernatant was retained and the pH was adjusted to pH 5.15 with dilute HCl. Stirring of the supernatant was continued at 4ºC for 15 min followed by centrifugation at 12100 x g for 10min at 4ºC. The supernatant was retained, to the supernatant 30% by volume of ice cold saturated $(NH_4)_2SO_4$ solution was added and the pH was adjusted to pH 4.6. Stirring of the supernatant was continued at 4ºC for 15 min followed by centrifugation at 12100 x g for 10min at 4ºC. The precipitate was resuspended in 5ml of RBA and stirred at 4ºC for 3 hours. The pH of the supernatant was adjusted to pH 5.7. Stirring of the supernatant was continued at 4ºC overnight followed by centrifugation at 12100 x g for 10min at 4ºC.

Step 2 (AMP affinity chromatography): A 5ml Bed volume AMP Sepharose column was prepared and equilibrated with 5 column volumes of Buffer A (10mM Imidazole, pH7; 10mM $MnCl_2$; 150mM NaCl). The *E.coli* GS supernatant from Step 1 was applied and

allowed to bind for 1 hour with gentle shaking at 4ºC. The column was washed with 10 column volumes of Buffer A. The *E.coli* GS was eluted with 12ml of Buffer B (10mM Imidazole, pH7; 10mM $MnCl_2$; 450mM NaCl; 2.5mM ADP). Fractions were collected and assayed using the λ-glutamyl transferase assay. The fractions with *E.coli* GS activity were pooled and dialysed overnight against RBA.

QC of isolated *E.coli* GS

SDS-PAGE analysis: The Laemmli method for sodium dodecyl-sulphate polyacrylamide gel electrophoresis (SDS-PAGE) was used to analyse the molecular mass and purity of the isolated enzyme (Laemmli, 1970). Gels were prepared as follows: 4% stacking gel (4% Bio-Rad Acrylamide-Bisacrylamide mix, 0.1% SDS, 0.05% ammonium persulphate, 0.1% TEMED, 0.05 M Tris-HCl, pH 6.8) and a 12% separating gel (12% Bio-Rad Acrylamide-Bisacrylamide mix, 0.1% SDS, 0.05% ammonium persulphate, 0.1% TEMED, 0.375 M Tris-HCl, pH 8.8). A 15 µl purified protein sample from each of the collected AMP affinity chromatography fractions (see enzyme purification above) was transferred to a clean microcentrifuge tube. Subsequently, an equal volume of denaturing buffer (1.2% SDS, 30% glycerol, 15% ß-mercaptoethanol, 0.18 mg/ml bromophenol blue, 0.15 M Tris, pH 6.8) was added. The protein samples were denatured at 90°C for 5 minutes. 10 µl of each sample was loaded onto the gel. Electrophoresis was performed in a 0.025 M Tris, 0.2 M Glycine buffer (pH 8.3) at 200 V in a Bio-Rad Mini Protean 3 Electrophoresis system. Protein bands were visualized with Coomassie Blue G250 staining solution (0.1 g Coomassie Blue G250 in 40% methanol, 10% acetic acid), and destaining solution (40% methanol, 10% acetic acid).

Protein concentration

Protein concentrations were determined by using the Quant-IT^{TM} Protein Assay Kit (Invitrogen, USA) that is used in conjunction with the QUBIT^{TM} fluorometer. The protein-containing sample is made up in 10µl, with the expected dilution, to this sample 190µl of the Quant-IT^{TM} working solution (1µl Quant-IT^{TM} reagent and 199µl Quant-IT^{TM} buffer) is

added. After a 15 minutes incubation time at room-temperature, the fluorescence is quantified by using the QUBIT™ fluorometer.

γ-glutamyl transferase assay for enzyme activity

This functional assay is carried out to: i) confirm the functionality of the purified TBGS enzyme; and ii) assess the degree of adenylylation of the purified enzyme.

The GS γ-glutamyl transferase enzyme activity is used to calculate the degree of adenylylation of GS using the standard method as outlined by Stadtman et al (1970). The basis of this assay is that at a specific pH the total enzyme activity of GS (both adenylylated and deadenylylated activity) occurs in the presence of Mn^{2+}. At the same pH in the presence of Mn^{2+} and an excess Mg^{2+}, only the deadenylylated component of the enzyme activity is measured. The resultant ratio is then used to calculate the degree of adenylylation of the enzyme.

HPLC analysis

Samples were analysed on a Hewlett Packard series 1100 HPLC fitted with a Luna 5µ C18 column. Each sample was automatically injected (2 µl) and separated with a mobile phase containing 51 mM KH_2PO_4, PIC A Low UV Reagent, 25% (v/v) acetonitrile. An AMP, ADP and ATP standard was used to calibrate the HPLC and the concentration of ADP in each sample was determined by the area under the curve using ChemStation software. The ADP values in the blank wells were subtracted from enzyme-containing wells, and percentage enzyme activity in each well calculated relative to the average net ADP values of the control wells without inhibitor.
